# PRKCSH serves as a potential immunological and prognostic biomarker in pan-cancer

**DOI:** 10.1038/s41598-024-52153-w

**Published:** 2024-01-20

**Authors:** Qiankun Wang, Xiong Wang, Jiaoyuan Li, Tongxin Yin, Yi Wang, Liming Cheng

**Affiliations:** Department of Laboratory Medicine, Tongji Hospital, Tongji Medical College, Huazhong University of Science and Technology, Wuhan, 430030 China

**Keywords:** Tumour biomarkers, Tumour immunology

## Abstract

Protein kinase C substrate 80K-H (PRKCSH) plays a crucial role in the protein N-terminal glycosylation process, with emerging evidence implicating its involvement in tumorigenesis. To comprehensively assess PRKCSH’s significance across cancers, we conducted a pan-cancer analysis using data from The Cancer Genome Atlas (TCGA), Genotype-Tissue Expression (GTEx), and Cancer Cell Line Encyclopedia (CCLE). We assessed aberrant PRKCSH mRNA and protein expression, examined its prognostic implications, and identified correlations with clinical features, tumor mutational burden (TMB), microsatellite instability (MSI), and tumor immunity across cancer types. We explored PRKCSH gene alterations, DNA methylation, and their impact on patient prognosis. Gene Set Enrichment Analysis (GSEA) and single-cell analysis revealed potential biological roles. Additionally, we investigated drug susceptibility and conducted Connectivity Map (Cmap) analysis. Key findings revealed that PRKCSH exhibited overexpression in most tumors, with a significant association with poor overall survival (OS) in six cancer types. Notably, PRKCSH expression demonstrated variations across disease stages, primarily increasing in advanced stages among eleven tumor types. Moreover, PRKCSH exhibited significant correlations with TMB in five cancer categories, MSI in eight, and displayed associations with immune cell populations in pan-cancer analysis. Genetic variations in PRKCSH were identified across 26 tumor types, suggesting favorable disease-free survival. Furthermore, PRKCSH methylation displayed a significant negative correlation with its expression in 27 tumor types, with a marked decrease compared to normal tissues in ten tumors. Cmap predicted 24 potential therapeutic small molecules in over four cancer types. This study highlights that PRKCSH, as a potential oncogene, may be a promising prognostic marker and therapeutic target of immunotherapy for a range of malignancies.

## Introduction

The incidence of cancer has significantly risen in recent decades, establishing itself as a major contributor to global human mortality^1^. According to the statistics from GLOBOCAN 2020, nearly 19.3 million individuals received new cancer diagnoses, resulting in 10.0 million cancer-related deaths^2^. Despite considerable efforts to advance cancer treatments, such as surgery, radiotherapy, chemotherapy, targeted therapy, and combination therapy, overall survival rates remain unfavorable and exhibit significant variability across different cancer types^3^. Extensive studies have centered on the tumor microenvironment (TME) and immunotherapy. Except for cancer cells, the TME encompasses a diverse array of elements, including tumor-related immune cells and other non-cellular components, all of which exhibit dual functions in both tumor promotion and suppression^4,5^. Cancer cells employ various mechanisms to evade immune surveillance and avoid elimination by tumor-suppressing immune cells, which include NK cells, M1-polarized macrophages, and effector T cells^5^. Cancer immunotherapy, particularly the use of immune checkpoint inhibitors (ICIs) therapy, aimed at harnessing the immune system to combat cancer, has achieved remarkable success in recent years^6^. Nevertheless, challenges persist, including the difficulty of predicting tumor immunotherapy response and the potential for adverse effects, impeding the broader clinical implementation of immunotherapy^7^. Consequently, there is an urgent need to identify more effective immune-related targets and predictive biomarkers of immunotherapy.

The glycosylation of protein is the process of covalently attaching polysaccharide chains to specific amino acid residues of protein polypeptide chains under the action of various glycosyltransferases and glycosidases, resulting in the formation of glycoproteins or proteoglycans^8^. This process is crucial for protein folding, quality control stability, and trafficking^8^. Glycosylated proteins are endowed with diverse functions such as cell–cell recognition, cell–matrix interactions, cell signaling, and maintenance of protein stability^9^. In the process of protein glycosylation, glycosyltransferases are mainly responsible for transferring sugar moieties onto proteins, while glycosidases play a role in degrading sugar chains. The two enzymes balance each other and jointly fine-tune the glycosylation process of proteins^9^. A large amount of research evidence has focused on the pivotal role of aberrantly glycosylated proteins in governing interaction, metabolism, immune escape and oncogenic signaling pathway within cancer cells^10^. Prominent clinical tumor markers, CA19-9, CA125, CEA, PSA, and AFP, are all products of aberrant glycosylation in malignant tumor cells. The dysregulated expression of glycosyltransferases and glycosylation is one of the important mechanisms for abnormal glycosylation of tumor cells^11^. PRKCSH, also referred to as glucosidase IIβ, interacts with glucosidase IIα to complete trimming of the terminal glucose residues of N-glycan, ensuring the quality control of glycoprotein folding within the endoplasmic reticulum (ER)^12^. Functioning as a noncatalytic subunit, glucosidase IIβassumes a critical role in glucosidase II localization within the ER and recognizing N-glycan^13^. The mutations in PRKCSH have the potential to lead to polycystic disease of the liver^14^.

Emerging evidence indicates that PRKCSH is involved in tumorigenesis and associated with poor prognosis in specific types of cancer. Elevated levels of PRKCSH expression are positively correlated to tumor stage and lymph node metastasis^15^. Previous studies have confirmed that inhibiting PRKCSH can induce autophagy and/or apoptosis in cancer cells through several pathways, such as the p53-dependent manner in lung carcinoma cells, mTOR-dependent pathway in Hela cells, and excessive ER stress in hepatocellular carcinoma^16–18^. In addition, knocking out PRKCSH has been shown to inhibit the growth and migration of lung cancer cells by disrupting receptor tyrosine kinase activities^19^. Moreover, Gu-Choul Shin et al. provided additional insight into the potential role of PRKCSH to drive tumorigenesis through its ability to enhance the inositol-requiring enzyme 1α signaling pathway and selectively confer resistance to ER stress in tumor-promoting factors^20^. More recently, it was reported that the lack of PRKCSH may activate STAT6 phosphorylation and p53 expression, resulting in G2/M arrest exposed to Nano-ZnO in lung cancer cells^19^.

Increasing evidence supports the significant role of abnormal protein glycosylation in evading immune surveillance, with the majority of tumor-associated carbohydrate antigens (TACAs) contributing to the immunosuppressive effects^21^. A notable example is that the immunosuppressive activity of PD-L1 is tightly regulated by N-terminal glycosylation^22^. However, despite the essential role of PRKCSH in the glycosylation process, its immunological significance in human cancer remains poorly understood. In this study, we performed a comprehensive pan-cancer analysis of PRKCSH, leveraging a range of public databases to investigate its differential expression, prognostic value, clinical correlates, TMB and MSI associations, tumor immune interactions, genetic alterations, DNA methylation patterns, biological functions, drug susceptibility profiles and Cmap analysis. This study aimed at exploring the prognosis predictive potential and tumor immunity function of PRKCSH in malignancies, offering insights into novel immunotherapy strategies.

## Materials and methods

### Data source and processing

The TCGA database (https://portal.gdc.cancer.gov/) provides gene expression data and clinical data for 33 tumors (Table 1). The TPM matrix, clinical information, and TMB data were downloaded using the TCGAbiolinks R package (v2.25.3) and its functions: GDCquery, GDCdownload, and GDCprepare^23^. PRKCSH expression profiles of tumor cell lines were obtained from the CCLE database (https://sites.broadinstitute.org/ccle/). The expression data underwent a log2(TPM + 1) transformation. The MSI data was acquired with the cBioPortalData R package (v2.6.1) by accessing studies from the cBio Cancer Genomics Portal (cBioPortal)^24^.

**Table 1:** Basic information of the 33 tumors and normal tissues.

TCGA cancer type	Detail	Normal	Tumor
ACC	Adrenocortical carcinoma	0	79
BLCA	Bladder urothelial carcinoma	19	409
BRCA	Breast invasive carcinoma	113	1113
CESC	Cervical squamous cell carcinoma and endocervical adenocarcinoma	3	306
CHOL	Cholangiocarcinoma	9	35
COAD	Colon adenocarcinoma	41	473
DLBC	Lymphoid neoplasm diffuse large B-cell lymphoma	0	48
ESCA	Esophageal carcinoma	13	185
GBM	Glioblastoma multiforme	5	169
HNSC	Head and neck squamous cell carcinoma	44	522
KICH	Kidney chromophobe	25	66
KIRC	Kidney renal clear cell carcinoma	72	538
KIRP	Kidney renal papillary cell carcinoma	32	291
LAML	Acute myeloid leukemia	0	151
LGG	Brain lower-grade glioma	0	534
LIHC	Liver hepatocellular carcinoma	50	374
LUAD	Lung adenocarcinoma	59	530
LUSC	Lung squamous cell carcinoma	51	501
MESO	Mesothelioma	0	87
OV	Ovarian serous cystadenocarcinoma	0	429
PAAD	Pancreatic adenocarcinoma	4	179
PCPG	Pheochromocytoma and Paraganglioma	3	184
PRAD	Prostate adenocarcinoma	52	502
READ	Rectum adenocarcinoma	10	167
SARC	Sarcoma	2	263
SKCM	Skin cutaneous melanoma	1	472
STAD	Stomach adenocarcinoma	36	412
TGCT	Testicular germ cell tumor	0	156
THCA	Thyroid carcinoma	59	513
THYM	Thymoma	2	120
UCEC	Uterine corpus endometrial carcinoma	35	550
UCS	Uterine carcinosarcoma	0	57
UVM	Uveal melanoma	0	80

### PRKCSH expression analysis

Initially, the ggpubr R package (v0.5.0) was utilized to analyze PRKCSH mRNA differential expression data from TCGA, comparing tumor and normal tissue. For non-paired sample comparisons, 23 tumor types with normal controls were included in the comparison in TCGA. It's worth noting that we restricted the analysis of paired samples to 15 tumors with a sample size exceeding 10, ensuring the accuracy and reliability of the results (Table 2). Secondly, the GEPIA2 database (http://gepia2.cancer-pku.cn/#analysis) was performed to analyze PRKCSH RNA sequencing data integrated from TCGA and GETx to affirm and complement the above analysis^25^. With the exception of UVM, which lacks normal controls in both GETx and TCGA, the remaining 9 types of tumors without normal controls in TCGA were included in this analysis. The ggplot2 R package (v3.3.3) was used to analyze the cell line expression matrix of tumors sourced from the CCLE dataset^26–28^. Thirdly, the UALCAN (http://ualcan.path.uab.edu/) was used to analyze PRKCSH protein expression levels within the Clinical Proteomic Tumor Analysis Consortium (CPTAC) and the International Cancer Proteogenome Consortium (ICPC) databases^29^. Additionally, the Human Protein Atlas (HPA, https://www.proteinatlas.org/search/PRKCSH) was used to retrieve the immunohistochemistry images of PRKCSH in both cancer and normal tissues. Moreover, the ROC curve was plotted using the pROC R package (v1.18.0) and the area under the curve (AUC) with 95% CI was presented to evaluate the diagnostic performance of PRKCSH.

**Table 2:** Basic information of the 15 tumors and paired normal tissues.

TCGA cancer type	Detail	Normal	Tumor
BLCA	Bladder urothelial carcinoma	19	19
BRCA	Breast invasive carcinoma	113	113
COAD	Colon adenocarcinoma	41	41
ESCA	Esophageal carcinoma	13	13
HNSC	Head and neck squamous cell carcinoma	43	43
KICH	Kidney chromophobe	25	25
KIRC	Kidney renal clear cell carcinoma	72	72
KIRP	Kidney renal papillary cell carcinoma	32	32
LIHC	Liver hepatocellular carcinoma	50	50
LUAD	Lung adenocarcinoma	58	58
LUSC	Lung squamous cell carcinoma	51	51
PRAD	Prostate adenocarcinoma	52	52
THCA	Thyroid carcinoma	33	33
THCA	Thyroid carcinoma	59	59
UCEC	Uterine corpus endometrial carcinoma	23	23

### PRKCSH correlates with prognosis and clinical features

The Cox regression analysis was utilized to evaluate the prognostic significance of PRKCSH in relation to OS for each cancer type using the survival (v3.4-0) and forestplot (v3.1.1) R packages. Each tumor type in the TCGA database was divided into low- and high-expression groups based on the median PRKCSH expression as the cut-off value, facilitating subsequent analysis. The survminer (v0.4.9) and survival (v3.4-0) R packages were implemented to perform a log-rank test and generate the Kaplan–Meier (KM) plot for data grouped according to PRKCSH expression level. Besides, the associations between PRKCSH expression and three clinical features including age at diagnosis, stage of the tumor, and gender of the patient were discussed separately in each tumor. This analysis was conducted by limma (v3.54.1) and ggpubr (v0.5.0) R packages.

### Correlation analysis on PRKCSH with TMB and MSI

TMB refers to the total number of nonsynonymous mutations per megabase in the exon-coding region of tumor cells^30^. Microsatellites are simple repeaters with a high degree of mutation in the genome, and MSI is defined as a microsatellite increases or decreases during DNA replication because of DNA mismatch repair (MMR) deficiency^31^. MSI can be classified as microsatellite stability (MSS) and high-frequency MSI (MSI-H). Elevated TMB and MSI levels are associated with increased neoantigen production by cancer cells and enhanced T cell recognition, clinically linked to improved outcomes with ICIs^30,31^. The relationships between PRKCHH expression with TMB/MSI were shown as radar plots, generated by the fmsb R package (v0.7.5).

Furthermore, to validate these findings, we categorized TMB into high and low groups within the samples of each TCGA tumor using the median as cutoff value. Subsequently, we conducted a t-test to compare PRKCSH gene expression between these two groups in each cancer type. Similarly, we segregated TCGA samples into MSS and MSI-H groups and compared PRKCSH gene expression between them.

### Relationship between PRKCSH expression of and immunity

Recognizing the intricate composition of cellular populations in the TME, Kosuke Yoshihara developed the ESTIMATE algorithm to estimate the abundance of stromal and immune cells within tumor samples using gene expression data^32^. The ESTIMATE algorithm computes immune score and stromal score used to depict the relative proportions of immune and stromal cells. The estimate store represents the combined percentage of the two ingredients. Firstly, the correlations of PRKCSH expression on the above scores of 33 tumors were analyzed with the estimate R package (v1.8.13). The Pearson correlation analysis was plotted using ggplot2 (v3.4.0) and ComplexHeatmap (v2.14.0) R packages. Secondly, the connections between immune infiltrating cells and PRKCSH expression were also recognized due to their important role in the TME. Furthermore, the relationship between immunotherapy-related molecules and the expression of PRKCSH was also investigated, including immune checkpoint-related genes, markers of immunostimulation and immunoinhibition, chemokines, and their receptors. The list of genes was downloaded from the TISIDB (http://cis.hku.hk/TISIDB/index.php). And the correlation analysis was performed using the ggplot2 R package (v3.4.0).

### Gene alteration, methylation, CNV and SNV analysis of PRKCSH

The cBioPortal (https://www.cbioportal.org/) was utilizedto gather genetic mutation details pertaining to PRKCSH based on TCGA Pan-Cancer Atlas data, including mutation frequency, mutation type, and mutation site. Moreover, the associations between the genetic alterations in PRKCSH and various clinical outcomes, including OS, disease-specific survival (DSS), progression-free survival (PFS), and DFS across pan-cancer were also investigated^24^.

The GSCA database (http://bioinfo.life.hust.edu.cn/GSCA/#/mutation) was performed to seek the differential methylation level of PRKCSH between tumor and corresponding normal tissues. Additionally, we assessed the relationship between methylation and expression levels of PRKCSH and its impact on patient prognosis. The copy number variation (CNV) and single-nucleotide variant (SNV) condition of PRKCSH were also identified in the same database^33^.

### Protein–protein interaction (PPI) network analysis and gene set enrichment analysis (GSEA)

GeneMANIA (https://genemania.org/) utilizes a vast amount of genomics and proteomics data to identify functionally similar genes^34^, and was used to build a protein–protein interaction (PPI) network of PRKCSH. We then grouped each type tumor in the TCGA database into low- and high-expression groups based on the median of PRKCSH expression and subsequently used the limma R package (v3.54.1) to identify differentially expressed genes (DEGs) between these groups. Furthermore, we conducted enrichment analyses of Gene Ontology (GO) and Kyoto Encyclopedia of Genes and Genomes (KEGG) on each cancer type using the org.Hs.eg.db (v3.16.0) and clusterProfiler (v4.6.2) R packages^35–37^. Finally, we presented the top five enriched terms using the enrichplot R package (v1.18.3).

### Single-cell analysis of PRKCSH

The CancerSEA database (http://biocc.hrbmu.edu.cn/CancerSEA/), which devotes to deciphering the functional states of cancer cells at the individual cell level, was utilized to explore the average correlation between PRKCSH and 14 functional states in 93,475 cancer single cells of 27 human cancer types. The threshold was set at a correlation strength of 0.3^38^.

### Assessment of drug susceptibility and potential compounds

The Gene Set Cancer Analysis (GSCA) database (http://bioinfo.life.hust.edu.cn/GSCA/#/), which integrates mRNA expression data and drug sensitivity data from the Genomics of Drug Sensitivity in Cancer database (GDSC) and the Cancer Therapeutics Response Portal database (CTRP), was used to perform a drug sensitivity analysis of PRKCSH in pan-cancer^33^.

The Connectivity Map (Cmap; https://clue.io/) database contains millions of gene expression profiles from different cell lines treated with bioactive small molecules, which can connect genes, drugs and disease states and help find potential small molecule drugs^39,40^. In this study, the 150 main up- and downregulated DEGs of PRKCSH for each cancer type were subjected to Cmap analysis in the “Latest” version and compounds with norm_cs < − 1.80 were identified as potential drugs for PRKCSH treatment.

### Statistical analysis

To analyze expression differences, we employed either paired t-tests or independent t-tests to compare PRKCSH expression levels between cancer and normal tissues, depending on the pairing of samples. We conducted comparisons of continuous variables among more than two groups using analysis of variance (ANOVA)^40–42^. The correlation coefficient between variables was determined by employing either Pearson or Spearman coefficients. A statistically significant difference was considered when P < 0.05. All statistical analyses and visualization were performed in R (v4.2.3).

## Results

### PRKCSH gene expression in pan-cancer patients

**Figure 1:**
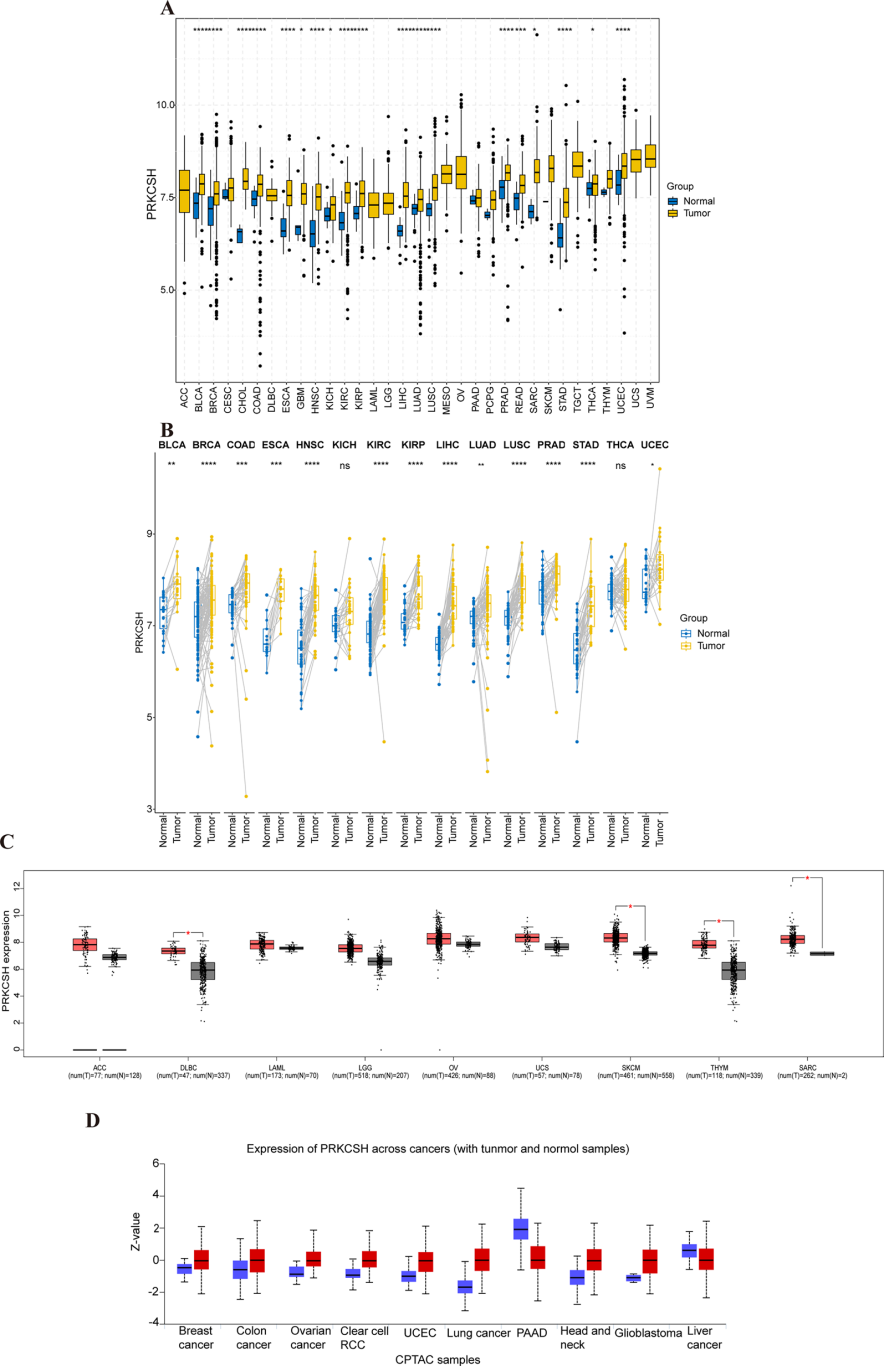
Pan-cancer PRKCSH expression levels. (**A**) The differences of PRKCSH mRNA expression between tumor and normal samples in 33 cancers based on TCGA. (**B**) Analysis of PRKCSH mRNA expression between tumor samples and paired non-tumor normal specimens using data from TCGA. (**C**) Expression levels of PRKCSH mRNA in ACC, DLBC, LAML, LGG, OV, UCS, SKCM, THYM and SARC using data integration from TCGA and GETx. (**D**) The expression levels of PRKCSH protein in different tumors in the CPTAC database. Red and blue color represent cancerous and normal tissues, respectively. ns P > 0.05; *P < 0.05; **P < 0.01; ***P < 0.001.

To elucidate the potential function of PRKCSH across different cancer types, we examined its mRNA expression levels in pan-cancer cohort. As presented in Fig. 1A, PRKCSH mRNA expression was significantly elevated in 19 types of tumors compared to their normal tissues among 23 tumor types in TCGA. Additionally, tumor tissues of BLCA, BRCA, COAD, ESCA, HNSC, KIRC, KIRP, LIHC, LUAD, LUSC, PRAD, STAD, and UCEC exhibited upregulated PRKCSH mRNA expression relative to their matched peritumoral tissues in the paired-samples analysis of 15 tumor types (Fig. 1B). Due to the lacking of normal controls for specific tumors in the TCGA database, we integrated TCGA and GETx data to analyze PRKCSH expression using the GEPIA2 database and found that PRKCSH was also up-regulated in DLBC, SKCM, THYM, and SARC (Fig. 1C). Furthermore, our analysis of CCLE data demonstrated distinct PRKCSH mRNA expression levels in 32 tumor cell lines, with the THCA cell line exhibiting the highest levels and the DLBC cell line displaying the lowest levels (Supplementary Fig. 1A–H).

**Figure 2:**
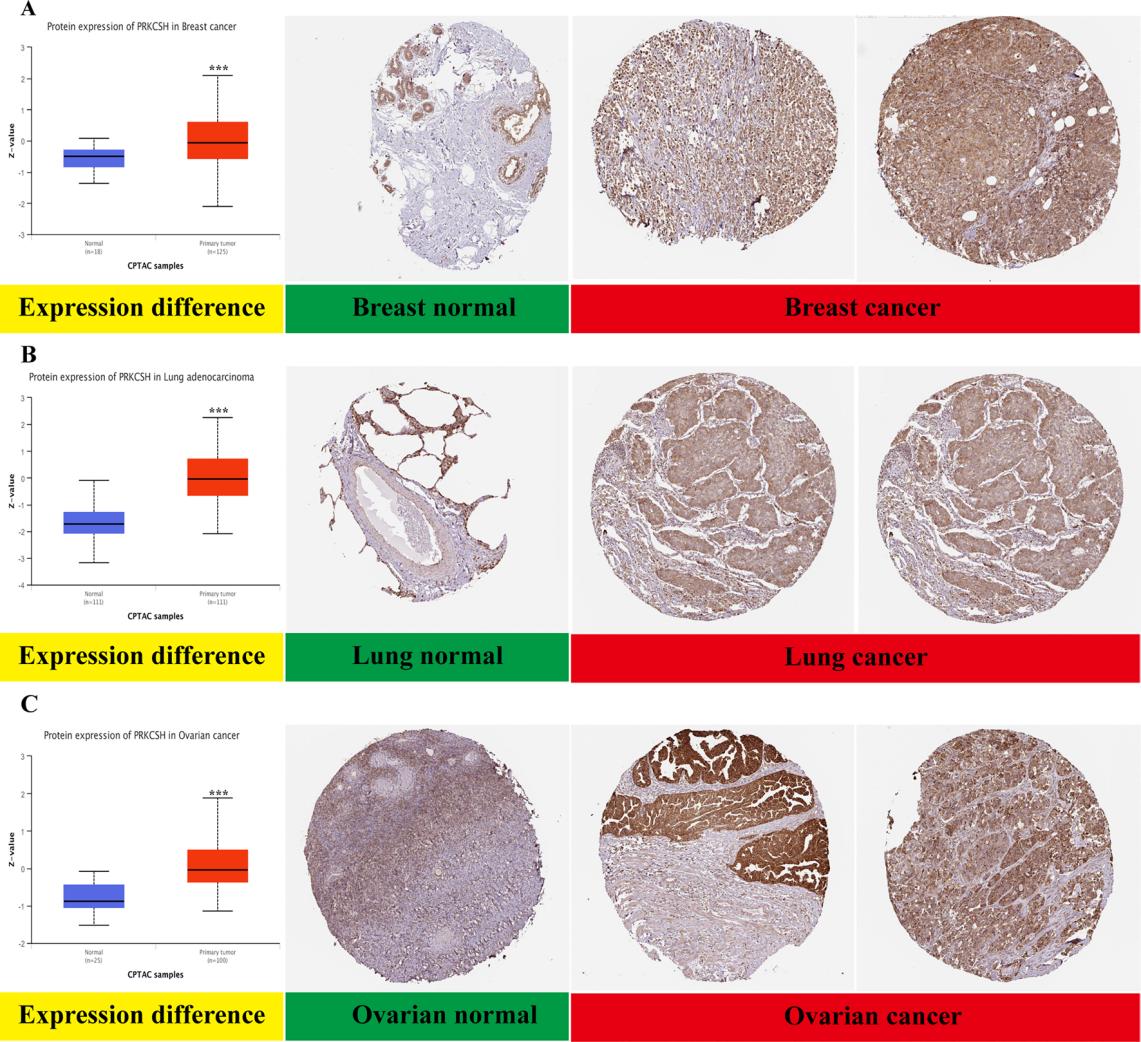
The PRKCSH protein differential expression between non-tumor normal tissues and tumor tissues from CPTAC (left) and immunohistochemistry images of PRKCSH in normal tissues (middle) and cancer tissues (right) obtained from HPA datasets. ***P < 0.001.

It is important to note that gene protein expression does not always coincide with its mRNA expression level. Therefore, we utilized the CPTAC database to compare and analyze the difference of PRKCSH protein expression between tumor and normal samples. The findings revealed that primary breast cancer, colon cancer, ovarian cancer, clear cell RCC, UCEC, lung cancer, head and neck squamous carcinoma, and glioblastoma exhibited higher levels of the total PRKCSH protein. However, surprisingly, PAAD and liver cancer presented a lower level of PRKCSH protein expression (Fig. 1D). The inconsistent levels of mRNA and protein expression in patients with PAAD and liver cancer may stem from an unidentified post-transcriptional regulatory mechanism that hinders the translation of PRKCSH mRNA. Additionally, immunohistochemical staining of the HPA database also showed that PRKCSH was highly expressed in most of the malignant tumors, and representative images were shown in Fig. 2A–C.

In addition, PRKCSH mRNA expression can distinguish well between cancerous and non-cancerous tissues. The AUC values were greater than 0.8 in eight types of tumor types, including BLCA, ESCA, HNSC, KIRC, LIHC, LUSC, READ, and STAD (Supplementary Fig. 2A–H). Notably, PRKCSH demonstrated the best diagnostic sensitivity and specificity in LIHC (AUC = 0.954). For the remaining nine tumors, except KICH, PAAD and THCA, the AUC values were above 0.7 (Supplementary Fig. 3A–I).

Taken together, these findings signify a significant increase in PRKCSH expression in the majority of cancers, and this consistency is observed at both the gene and protein levels, laying the groundwork for further investigation.

### The prognostic value of PRKCSH in pan-cancer

**Figure 3:**
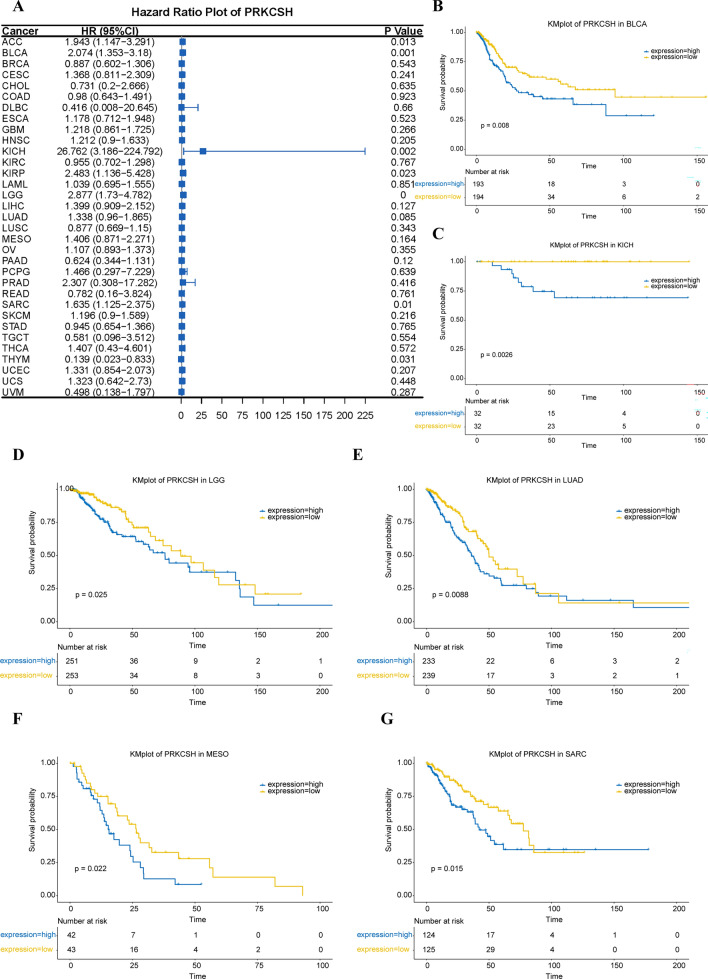
The relationship between PRKCSH expression and OS. (**A**) Forest map of the relevance of PRKCSH expression with OS of 33 kinds of tumors in the TCGA cohort. (**B**–**G**) Kaplan-Meir curves showing the association between PRKCSH expression and patients’ OS of (**B**) BLCA, (**C**) KICH, (**D**) LGG, (**E**) LUAD, (**F**) MESO and (**G**) SARC based on PRKCSH expression.

To further explore the prognostic value of PRKCSH, we then used clinical data from the TCGA database for survival analysis. The Cox analysis identified PRKCSH as a high-risk gene in ACC, BLCA, KICH, KIRP, LGG, and SARC (Fig. 3A). KM survival analysis indicated that elevated PRKCSH expression implied poor OS of BLCA, KICH, LGG, LUAD, MESO, and SARC (Fig. 3B–G). Consequently, higher PRKCSH expression in tumor patients suggests an unfavorable prognosis.

### Correlation between expression of PRKCSH and clinical characteristics in different tumors

Then, we examined the differential expression of PRKCSH based on the age at diagnosis. Higher PRKCSH expression was observed in CHOL, GBM, and THYM patients aged > 60 years. In the contrast, in KIRP, individuals over 60 had lower levels of PRKCSH expression than patients who were younger than 60 (Supplementary Fig. 4A–D).

Subsequently, we examined the relationship between the PRKCSH expression and tumor stage. The results revealed significant differences between PRKCSH expression and tumor stage in ACC, COAD, HNSC, KICH, KIRC, KIRP, LUAD, LUSC, MESO, UCEC, and UCS (Supplementary Fig. 5A–K). PRKCSH was generally more expressed in higher tumor stages (stage III or IV vs. I or II), especially in ACC (stage III vs. I, stage IV vs. I, stage III vs. II, stage IV vs. II) and KICH (stage II vs. I, stage IV vs. I, stage IV vs. II, stage IV vs. III).

Furthermore, we also explored potential gender-based differences in PRKCSH expression among . As illustrated in Supplementary Fig. 6, male patients with HNSC, PAAD, and STAD exhibited higher PRKCSH expression, while female KIRP patients displayed a similar pattern.

### Association of PRKCSH expression with TMB and tumor MSI

**Figure 4:**
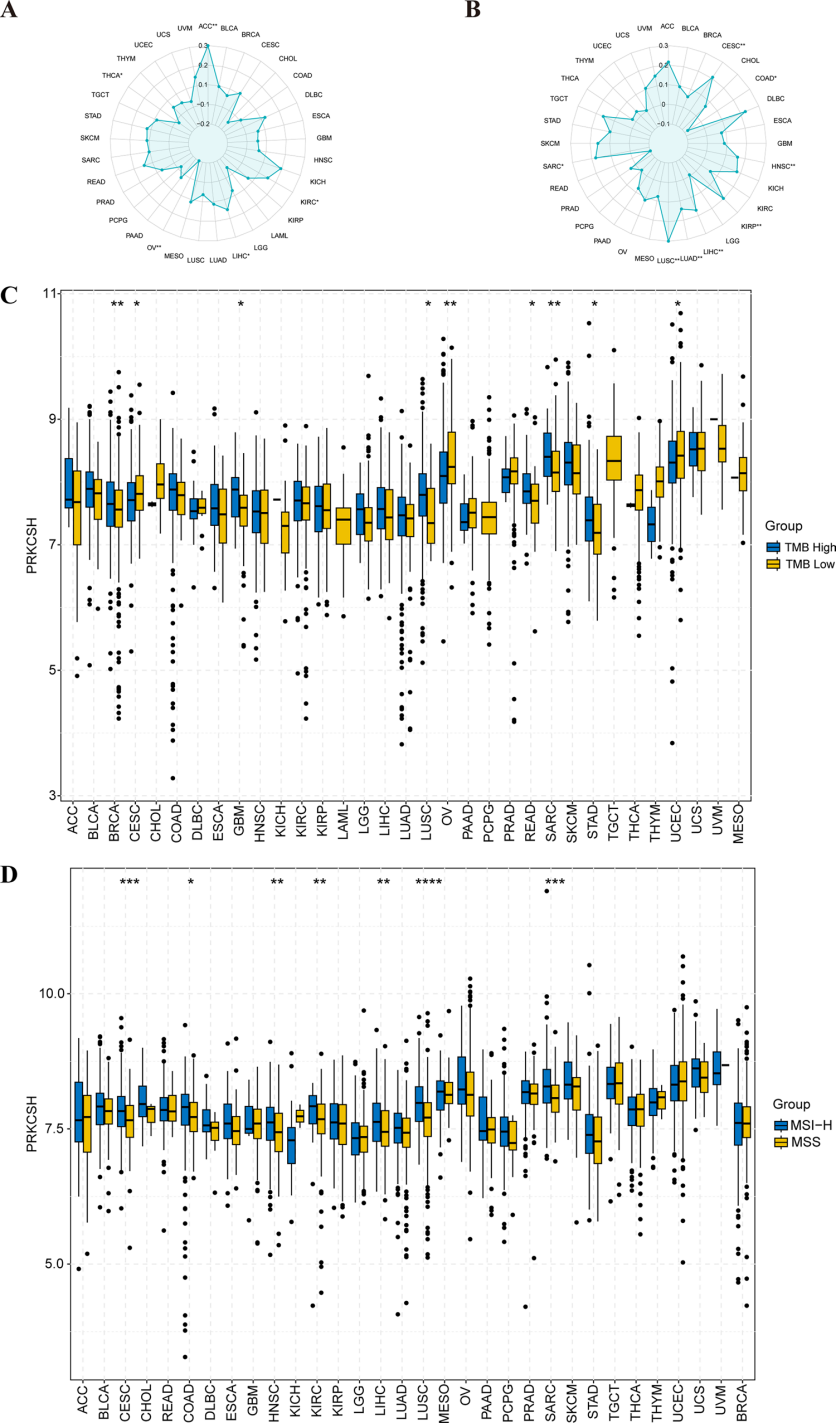
Radar diagram demonstrating the correlation of PRKCSH with (**A**) TMB and (**B**) MSI. (**C**) Differences in PRKCSH expression between the TMB high and TMB low groups. (**D**) Differences in PRKCSH expression between the MSS and MSI-H groups. *P < 0.05, **P < 0.01, and ***P < 0.001.

Giving the significance of TMB and MSI in predicting immunotherapy outcomes, we inspected the relationships between PRKCSH expression and these two indicators. PRKCSH expression was positively correlated with TMB in ACC, KIRC, and LIHC. Whereas it exhibited an inverse correlation in OV and THCA (Fig. 4A). Additionally, PRKCSH expression was positively correlated with MSI in CESC, HNSC, KIRP, LIHC, LUAD, LUSC, and SARC but negatively in COAD (Fig. 4B).

Additionally, we conducted a comparison of PRKCSH gene expression between the TMB high and TMB low groups (Fig. 4C). The findings indicated that PRKCSH expression was higher in the TMB high group in BRCA, GBM, LUSC, READ, SARC, STAD, and UCEC. Conversely, in the case of OV and CESC, the opposite pattern was observed. Similarly, the expression of PRKCSH was higher in the MSI-H group in CESC, COAD, HNSC, KIRC, LIHC, LUSC and SARC aligning with the correlation trends mentioned above (Fig. 4D).

While ICIs represent promising cancer immunotherapies and have seen clinical use, their effects are regrettably limited and may lead to various unique immune-related toxicities and accelerated disease progression, which highlights the significance of identifying predictive biomarkers^43^. Our findings provided evidence that abnormal expression of PRKCSH can affect TMB and MSI and further affect immunotherapy response.

### Expression of PRKCSH is related to TME

**Figure 5:**
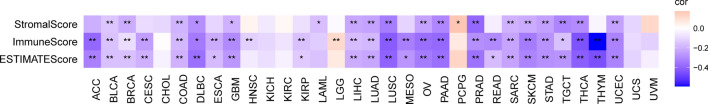
A heatmap of associations of PRKCSH expression with StromalScore, ImmuneScore and ESTIMATEScore across different types of cancer. *P < 0.05, **P < 0.01, and ***P < 0.001.

To assess the connection between PRKCSH expression and the TME, we analyzed the immune score, stromal score, and estimate score in 33 tumors with the estimate R package (v1.0.13). The resulting heatmap illustrated that PRKCSH displayed a negative correlation with the above three scores in most tumors except for CHOL, KICH, KIRC, UCS, and UVM. Particularly, PRKCSH was positively linked to the immune score in LGG and with the stromal score in PCPG (Fig. 5).

### Link between PRKCSH expression and tumor-infiltrating immune Cells

**Figure 6:**
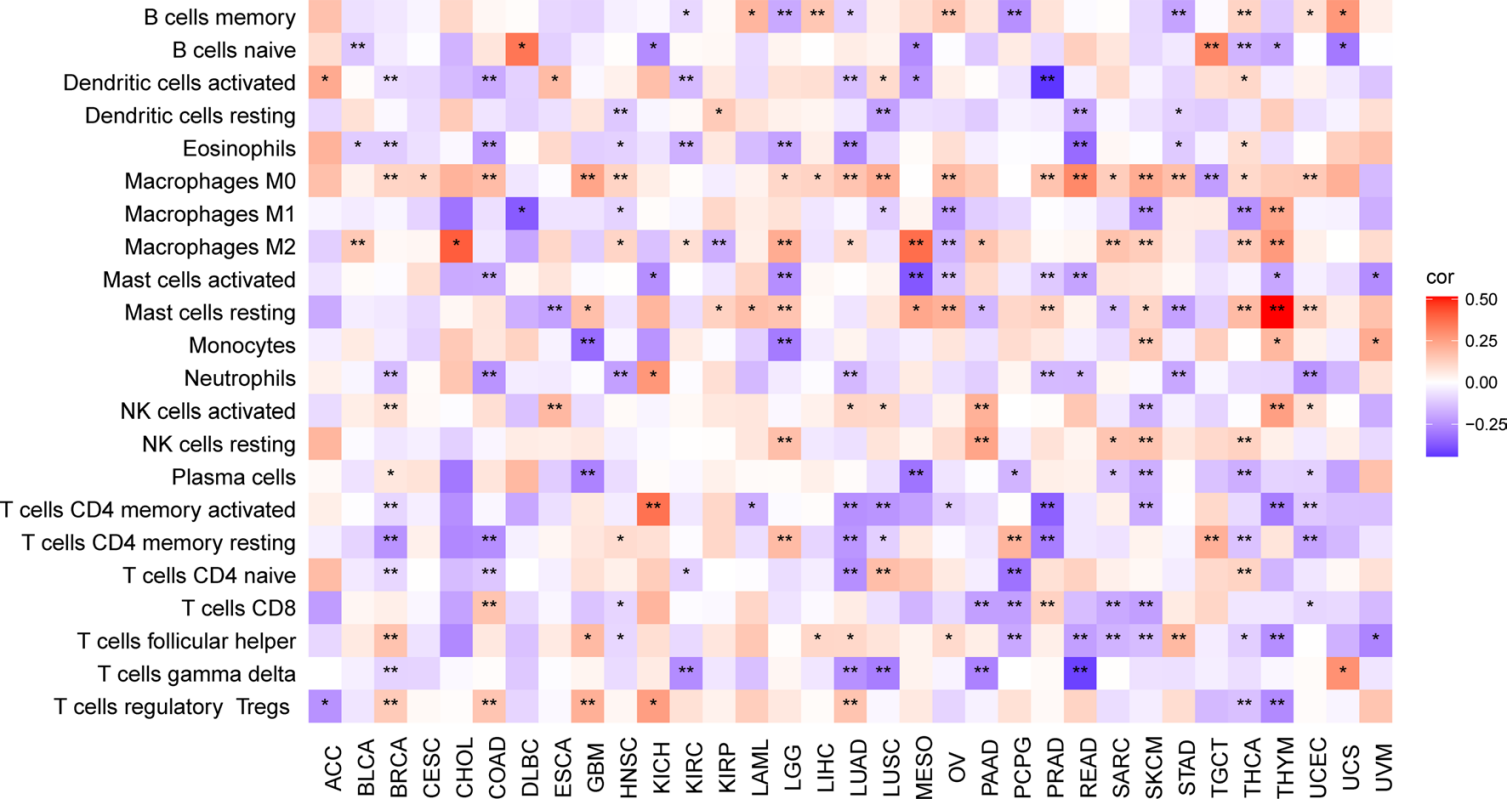
Association of PRKCSH expression with tumor-infiltrating immune cells. *P < 0.05, **P < 0.01, and ***P < 0.001.

Tumor-infiltrating immune cells are widely recognized for their crucial role in tumor progression and evasion of the immune response. Thus, we analyzed the correlation between the PRKCSH level and the infiltration levels of 22 non-tumor cells in the TME. Our findings revealed a strong correlation between PRKCSH expression and the immune cells in pan-cancer (Fig. 6).

**Figure 7:**
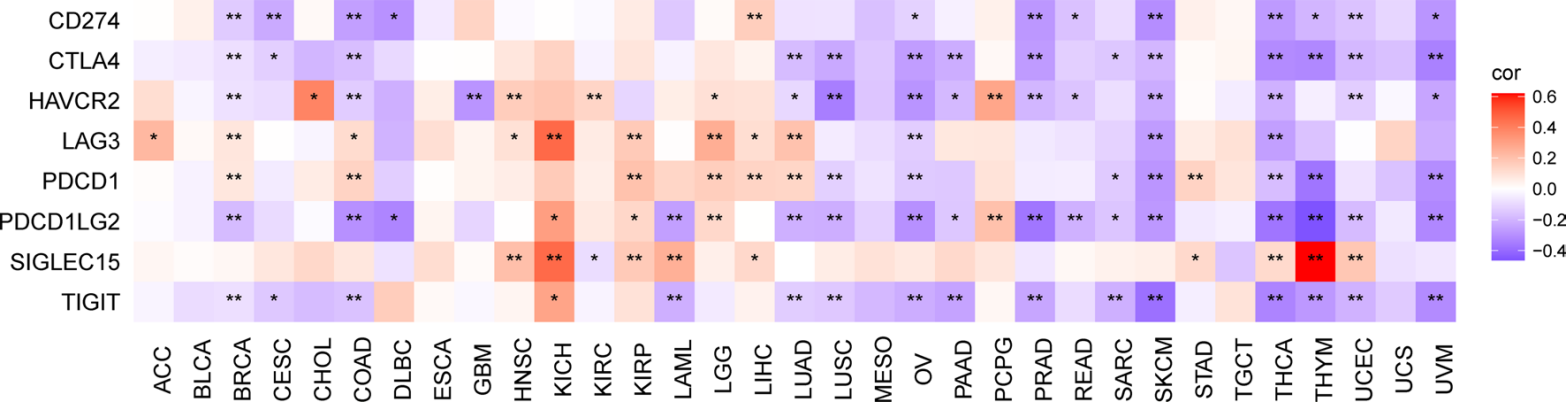
A heatmap about the association between PRKCSH expression and checkpoint-associated genes. *P < 0.05, **P < 0.01, and ***P < 0.001.

Immune checkpoints play indispensable regulatory roles in tumor immune escape, serving to shield tumor cells from surveillance and destruction by the immune system. Therefore, we examined the relationship between PRKCSH expression and eight immune checkpoint genes, and the results showed that PRKCSH expression levels were generally negatively correlated with these genes in most tumors except for HNSC, KICH, KIRC, KIRP, LGG, LIHC, PCPG, and STAD, as depicted in Fig. 7. Overall, these results suggest the potential role for PRKCSH in tumor immunity regulation.

### PRKCSH expression correlates with immune molecules in pan-cancer

**Figure 8:**
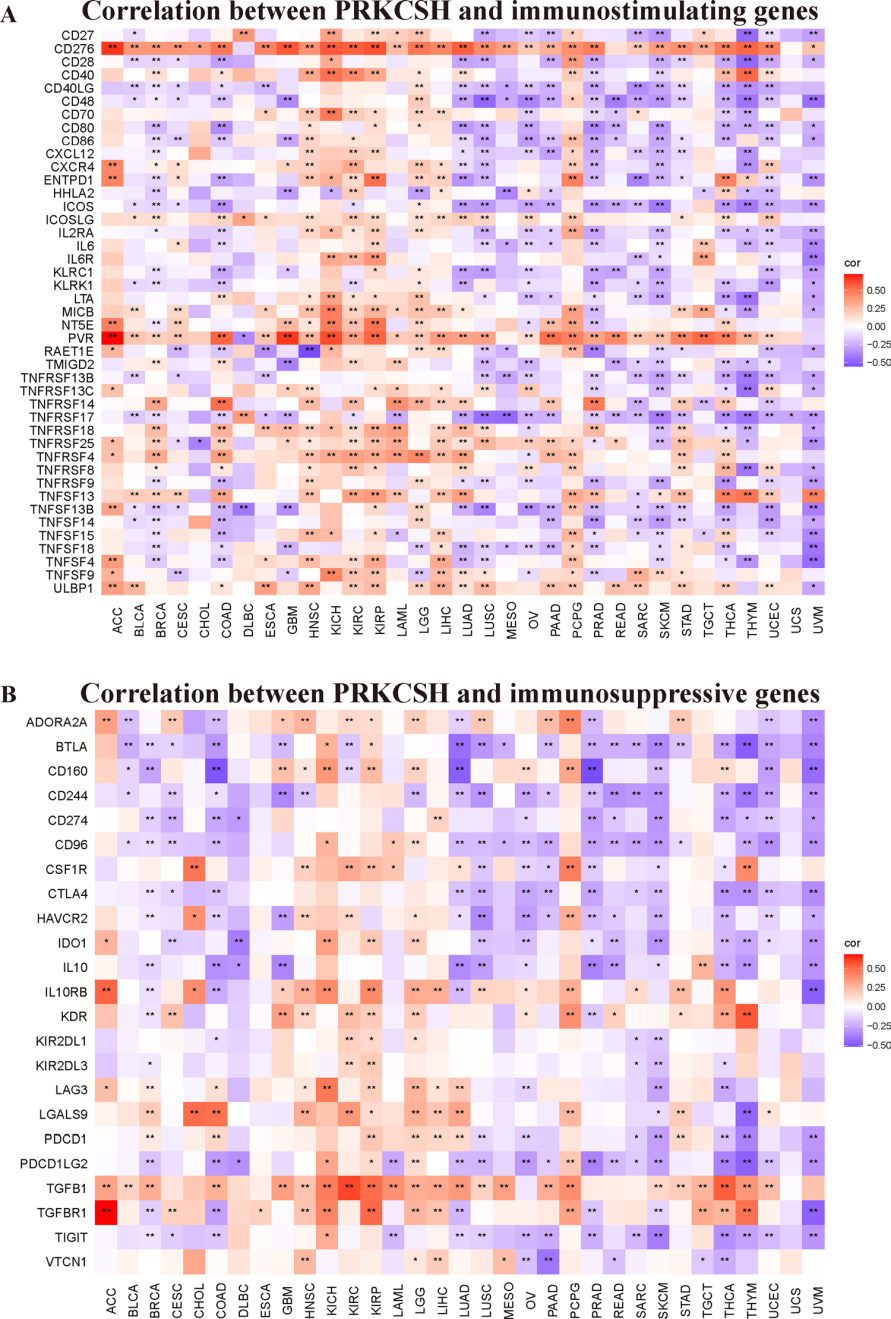
The relation of PRKCSH to (**A**) immunostimulating and (**B**) immunosuppressive genes. *P < 0.05, **P < 0.01, and ***P < 0.001.

**Figure 9:**
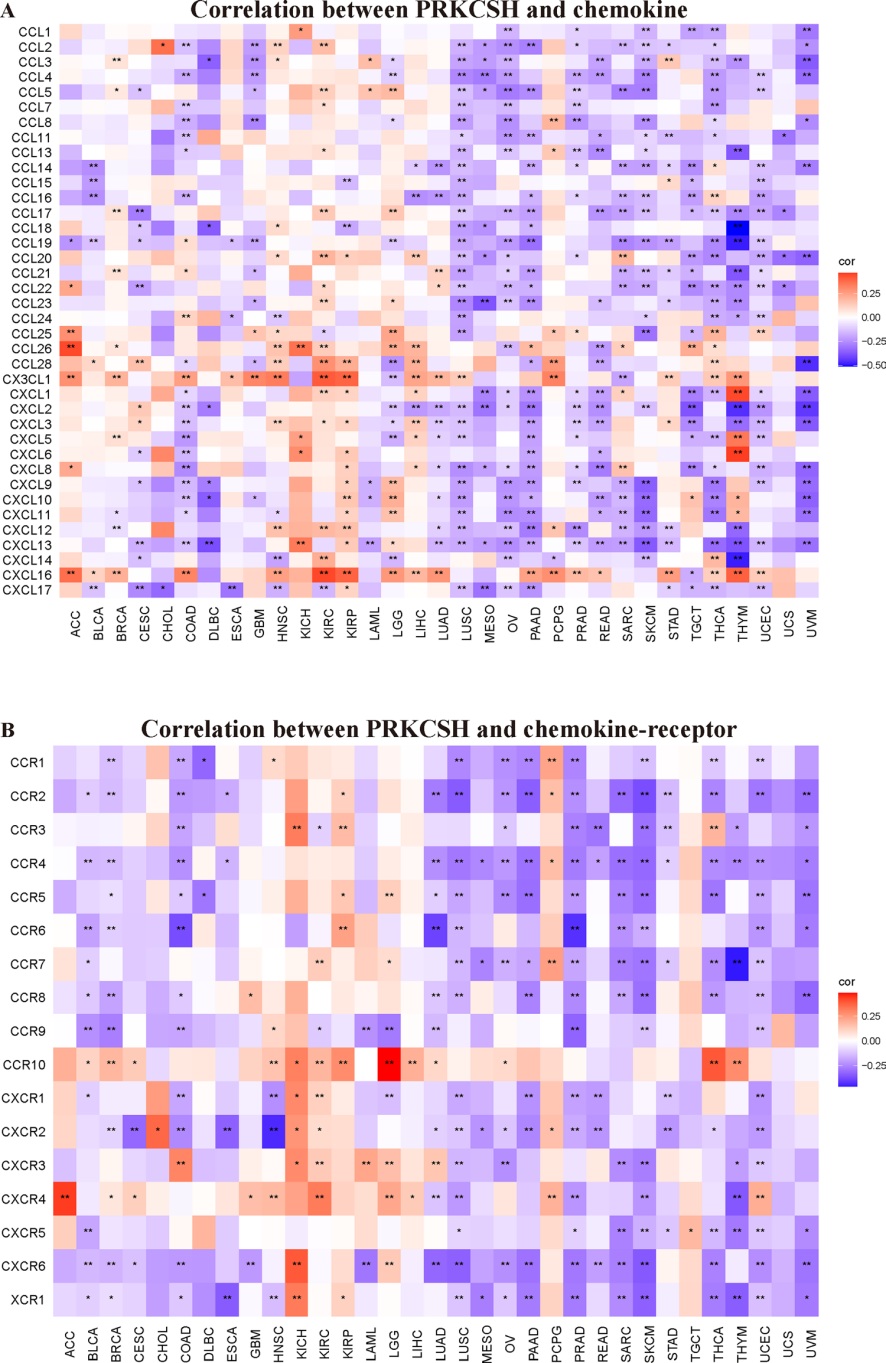
Correlation between PRKCSH expression with (**A**) chemokine genes and (**B**) chemokine-receptor genes. *P < 0.05, **P < 0.01, and ***P < 0.001.

Then, we sought the links between PRKCSH expression and a variety of immune signatures to enlarge the comprehension of the function of PRKCSH in tumor immunity. Figure 8A,B show the connection of PRKCSH with the immunostimulator and immunoinhibitory genes, respectively. As a result, PRKCSH was negatively correlated with most of the immunostimulatory genes, except for CD276, ICOSLG, PVR, TNFSF13, and ULBP1 (Fig. 8A). As for immunoinhibitor factors, PRKCSH showed a negative correlation with BTLA, CD244, CTLA4, CD274, and IL10 in most of the tumors, except in ACC, CHOL, HNSC, KICH, KIRC, KIRP, LGG, LIHC, PCPG, and TGCT (Fig. 8B). Next, we examined the chemokines and their receptors which may be regulated by PRKCSH. The results revealed negative relationships between most of the chemokines and their receptors and PRKCSH. The chemokines which highly correlated with PRKCSH were CXCL13, CXCL16, CX3CL1, and CXCL2 (Fig. 9A), and the receptors which highly associated with PRKCSH were CXCR6, CCR4, CCR2, and CXCR2 (Fig. 9B).

### The genetic variation, methylation, CNV, and SNV information of PRKCSH

**Figure 10:**
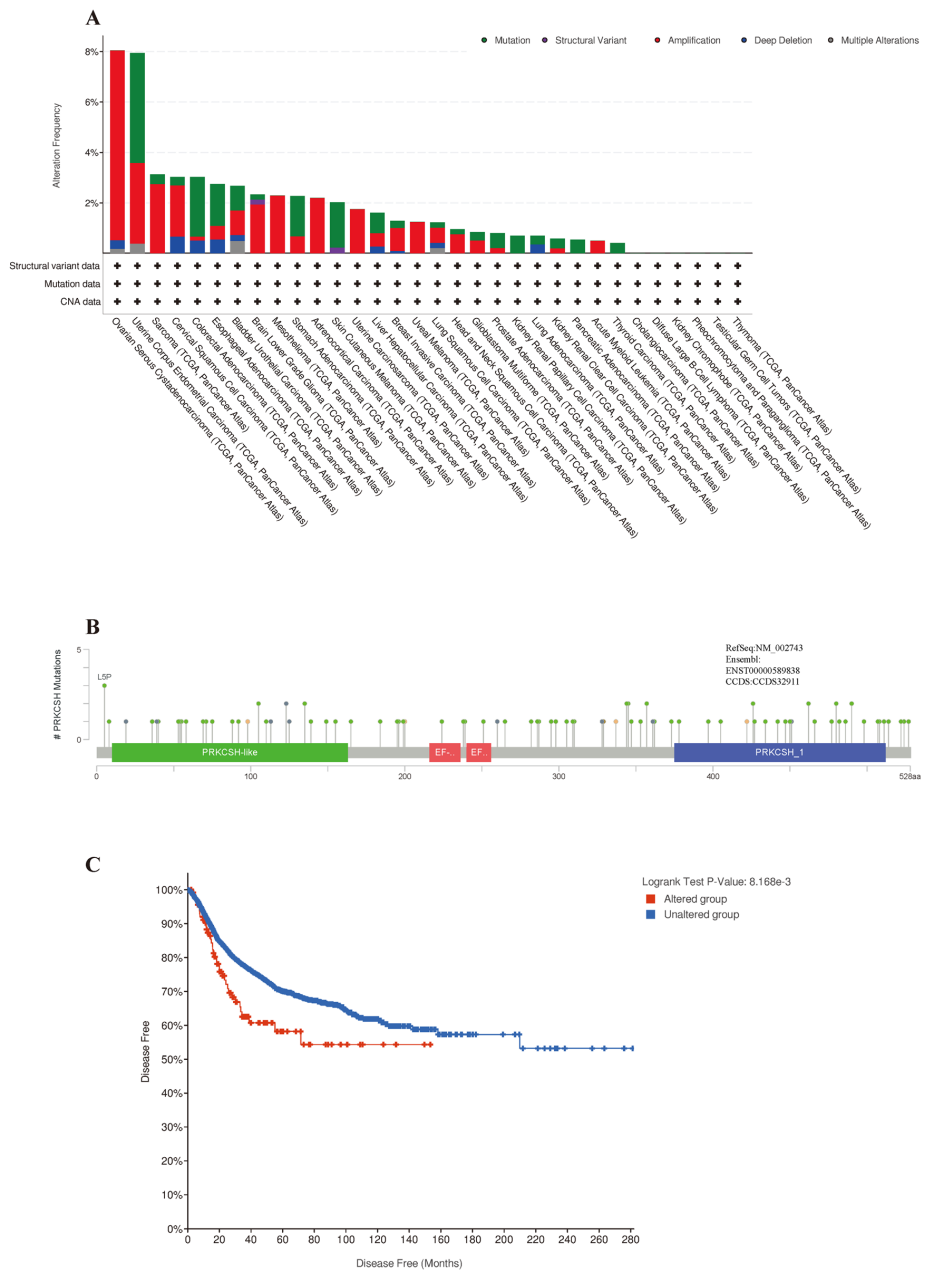
Genetic alteration analysis of PRKCSH by cBioPortal. (**A**) The alteration frequency distribution of different mutation types of PRKCSH in different tumors. (**B**) Presentation of the types, sites, and case number of PRKCSH genetic alteration across protein domains. (**C**) Patients without the alteration of PRKCSH had a better prognosis in DFS.

Then we explored the potential mechanisms underlying the high expression of PRKCSH mRNA in tumor cells. On the one hand, the role of genetic mutation in driving cancer has been extensively reported. We next searched the cBioPortal to gain insights into the structure and gene alterations of PRKCSH. As shown in Fig. 10A, patients with OV exhibited the highest rate of PRKCSH mutations (about 8%), with “amplification” as a primary alteration type. Furthermore, Fig. 10B summarizes the types, sites, and case numbers of PRKCSH genetic alteration. We found that missense mutations are the main type of mutation in PRKCSH, with L5P alteration detected in one case of astrocytoma, one case of uterine Endometrioid, and one case of colon adenocarcinoma. Subsequently, we explored the potential association between PRKCSH alteration and survival outcomes in pan-cancer. The results, as illustrated in Fig. 10C, indicated that PRKCSH-altered patients exhibited an improved prognosis in DFS but not in OS, PFS, or DSS when compared with PRKCSH-unaltered cases.

CNV and SNV are both genetic variations with distinct characteristics. CNV is a general term used to describe a repeated DNA segment of 1 kb or more, which can result in a coding gene dosage variation^44^. SNV presents as either germline or somatic point mutations and may affect folding, binding affinity, expression, post-translational modification, and other protein functions^45^. The CNV and SNV of PRKCSH were analyzed using the GSCA platform. The distribution of CNV percentages across different cancer types is shown in Supplementary Fig. 7A. We observed that the CNV of PRKCSH was particularly high in patients with ACC, SARC, OV, LUSC, ESCA, UCS, LUAD, and TGCT. The CNV of ACC, KICH, SARC, GBM, PCPG, and LGG patients was mainly heterozygous amplification, while patients with TGCT, LUAD, ESCA, LUSC, STAD, CESC, and BLCA are predominantly heterozygous deletion. In addition, we further explored the connection between the expression of PRKCSH mRNA and CNV, and found significant positive correlations in 18 tumors (Supplementary Fig. 7B). In terms of SNV, the proportion was notably higher in UCEC and COAD, accounting for the percentages of 22% and 12%, respectively (Supplementary Fig. 7C).

**Figure 11:**
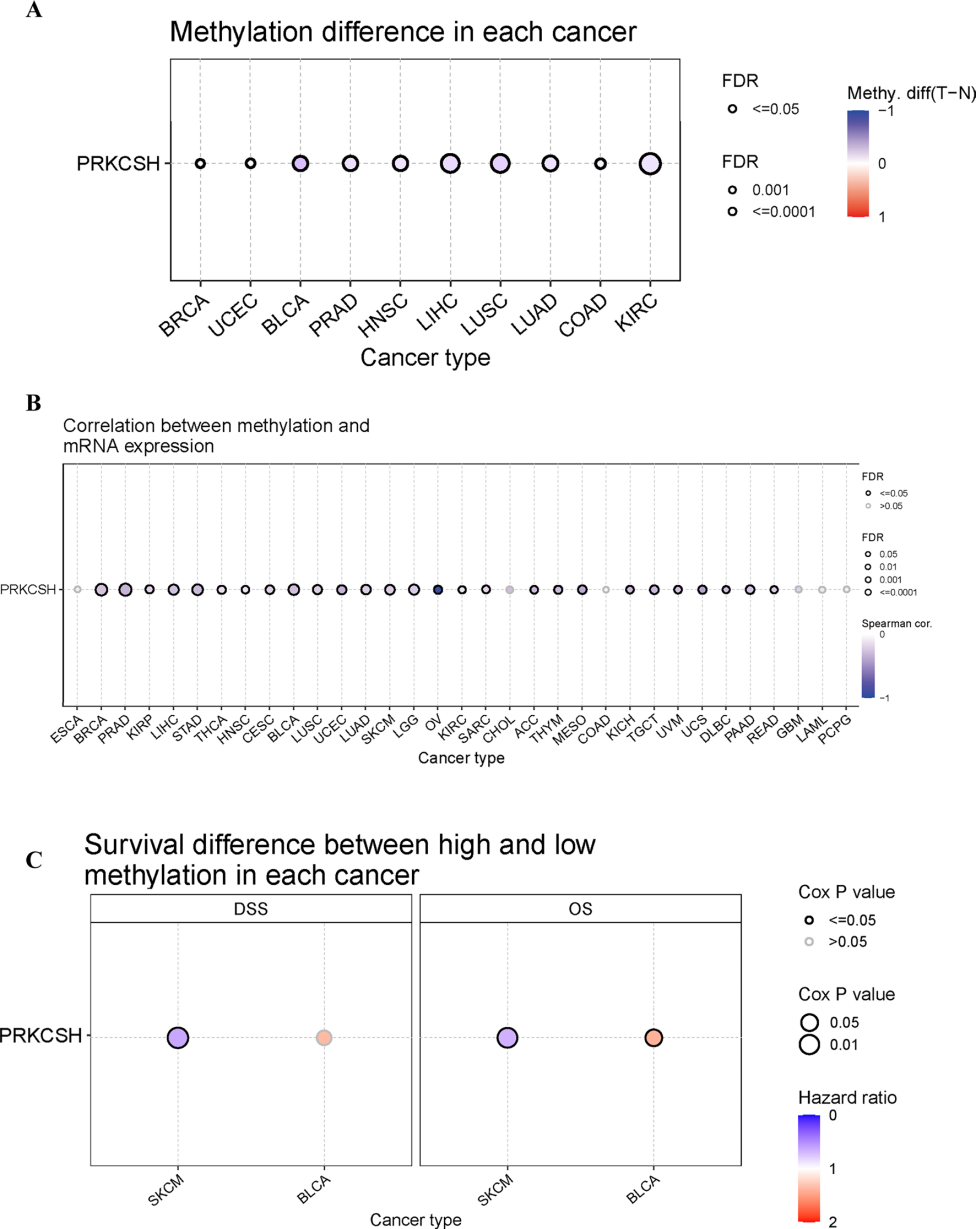
High expression of PRKCSH correlates with DNA hypomethylation and methylation-related survival. (**A**) Methylation difference of PRKCSH between tumor and normal samples in each cancer. (**B**) The association of DNA methylation of PRKCSH with its expression in the specific cancers. The circle size represents the FDR value, and the color shade represents the correlation coefficient. (**C**) The DNA methylation of PRKCSH is associated with DSS and OS in SKCM and BLCA.

Gene methylation, considered one of the most critical epigenetic modifications, exerts its influence by recruiting proteins involved in gene repression or by inhibiting the binding of transcription factors to DNA^46^. In the present study, we investigated the potential involvement of DNA methylation in PRKCSH mRNA expression using the GSCA database. In summary, the expression of PRKCSH was inversely correlated with its methylation level in 27 cancers, excluding ESCA, CHOL, COAD, GBM, LAML, and PCPG (Fig. 11A). Specifically, PRKCSH showed hypomethylation in patients with BRCA, BLCA, COAD, HNSC, KIRC, LIHC, LUAD, LUSC, PRAD, and UCEC (Fig. 11B). Moreover, survival analysis showed that the enhanced PRKCSH methylation was a protective factor for prognosis, leading to better OS and DSS in patients with SKCM. Conversely, in patients with BLCA, it was identified as a risk factor for OS (Fig. 11C).

This section suggested that gene mutations and hypomethylation may contribute to the abnormal expression of PRKCSH in tumors, potentially influencing prognosis.

### PPI network analysis and enrichment analysis based on PRKCSH expression

**Figure 12:**
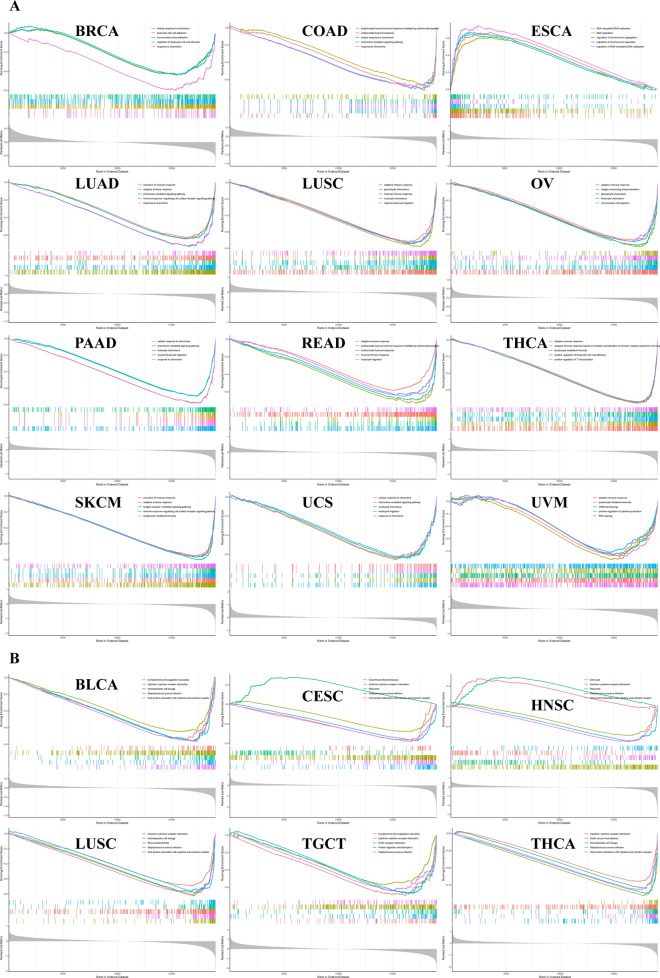
GSEA functional analysis of PRKCSH. (**A**) GO functional annotation of PRKCSH gene in BRCA, COAD, ESCA, LUAD, LUSC, OV, PAAD, READ, SKCM, THCA, UCEC, UCS, and UVM. (**B**) KEGG pathway analysis of PRKCSH gene in BLCA, LUSC, THCA, CESC, HNSC, and TGCT^32–34^.

To explore the function of PRKCSH and predict the underlying oncogenic mechanism of PRKCSH, the PPI network analysis and GSEA enrichment analysis were performed. Using GeneMANIA database, 20 co-expressed genes with PRKCSH were obtained from the (Supplementary Fig. 8), among which GANAB, MLEC and CALR exhibited the most significant correlation with PRKCSH. We then grouped cancer patients according to the expression level of PRKCSH and performed GSEA enrichment analysis on the differential genes to explore the biological function of PRKCSH in different cancers. GO analysis displayed that PRKCSH was significantly linked with the functions of adaptive immune response, response to chemokine, DNA replication, and humoral immune response (Fig. 12A). KEGG pathway analysis unveiled that PRKCSH was mainly involved in viral protein interaction with cytokine and cytokine receptors, staphylococcus aureus infection, hematopoietic cell lineage, and cytokine-cytokine receptor interaction (Fig. 12B).

### Single-cell function analysis of PRKCSH

**Figure 13:**
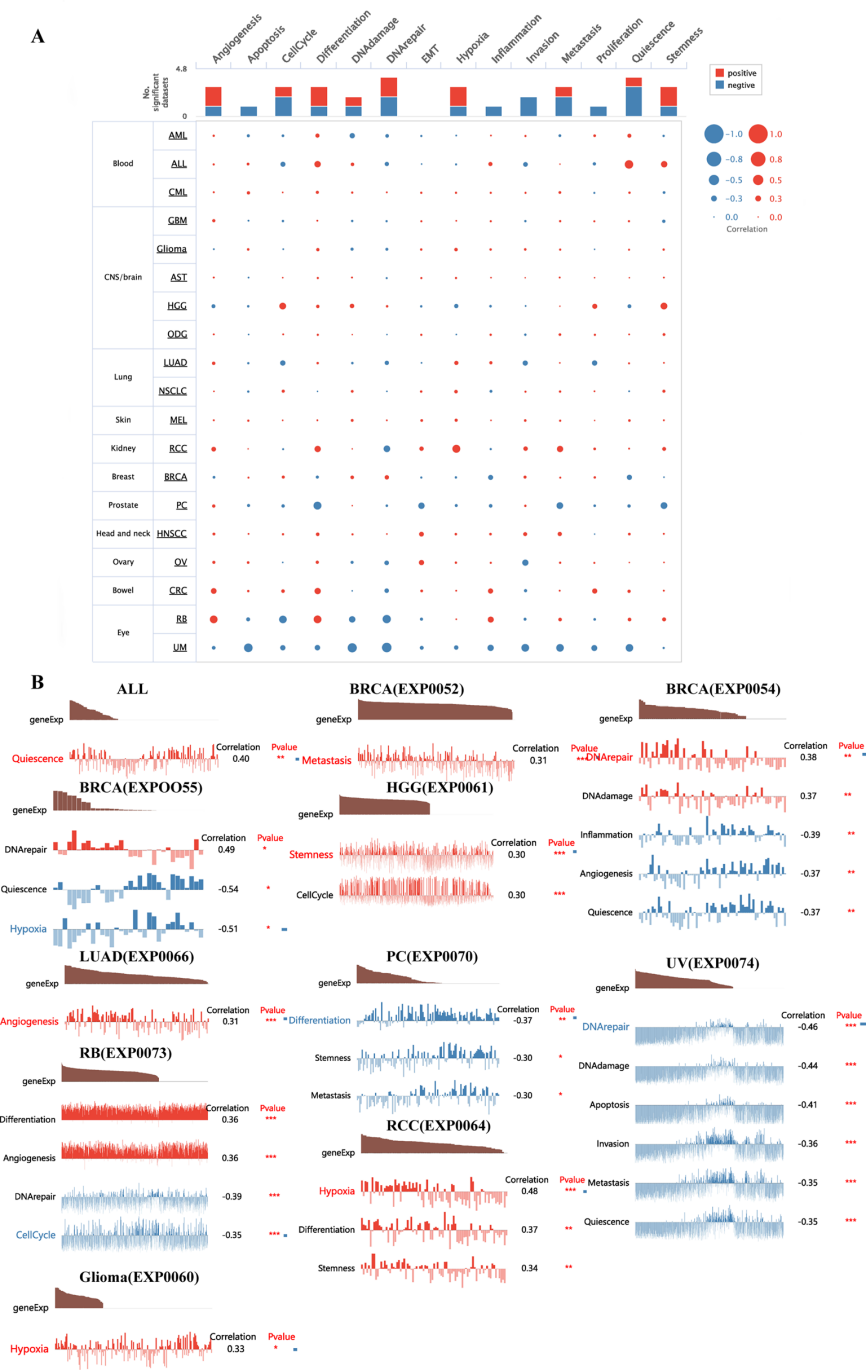
The single-cell analysis of PRKCSH. (**A**) Different functional status is related to PRKCSH in many cancers. (**B**) Correlation analysis between functional status and PRKCSH in ALL, BRCA, Glioma, HGG, LUAD, PC, RB, RCC, and UM. *P < 0.05, **P < 0.01, and ***P < 0.001.

Moving forward, we conducted an assessment of cancer-related functional states of PRKCSH at the single-cell sequencing level using the CancerSEA database. PRKCSH exhibited positive correlations with distinct cellular processes in different cancers, such as quiescence in acute lymphoblastic leukemia (ALL); with metastasis, DNA repair, and DNA damage in BRCA; with hypoxia in glioma; with stemness and cell cycle in high-grade glioma; with angiogenesis in LUAD; with differentiation and angiogenesis in retinoblastoma (RB); with hypoxia, differentiation and stemness in renal cell carcinoma (RCC). Conversely, PRKCSH displayed negative correlations with quiescence, inflammation, and angiogenesis in BRCA; with differentiation, stemness, and metastasis in prostate cancer (PC); with DNA repair, DNA damage, apoptosis, invasion, metastasis, and quiescence in UVM; with DNA repair and cell cycle in RB (Fig. 13A,B).

### Drug sensitivity and Cmap analysis of PRKCSH

We examined the impact of PRKCSH mRNA expression on drug susceptibility based on GSCA. Our findings revealed that high mRNA expression of PRKCSH was positively associated with the sensitivity of drugs including XAV939, indisulam, and tacedinaline, but inversely with the sensitivity of drugs including (5Z)-7-Oxozeaenol, PD-0325901, PLX4720, RDEA119, Trametinib, and selumetinib (Supplementary Fig. 9A,B).

**Figure 14:**
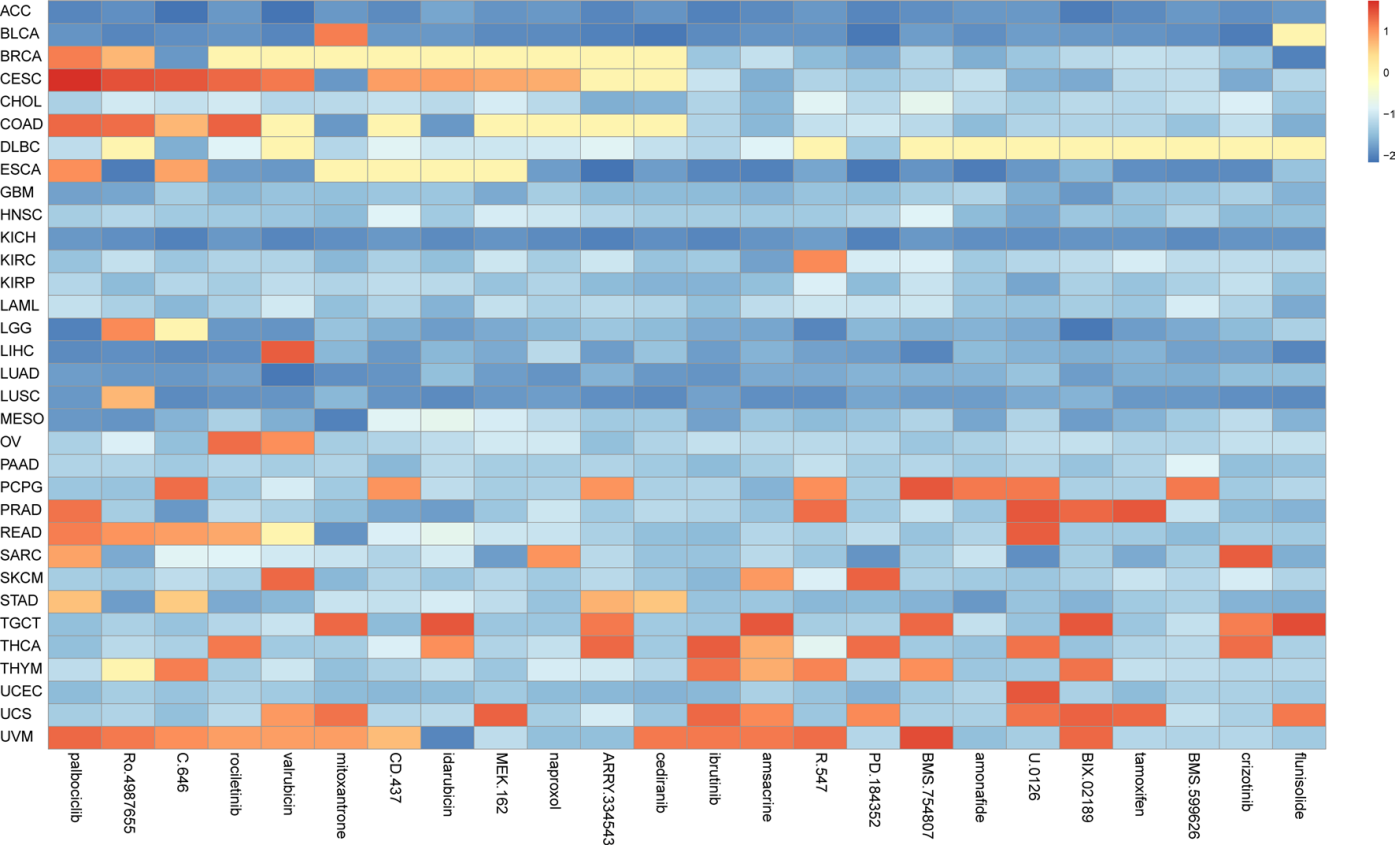
Heat map represents the norm_cs (blue negative, red positive) for each drug in the CMap database for each cancer.

Finally, the Cmap analysis identified 24 small-molecule drugs with the potential therapeutic efficacy targeting PRKCSH in more than 4 cancer types (Fig. 14). The heatmap depicts the norm_cs values of 24 small molecule drugs in pan-cancer. Palbociclib, Ro-4987655 and C-646 were significantly enriched in 8 cancers. Palbociclib, a cyclin-dependent kinase (CDK) 4/6 inhibitor approved for breast cancer treatment, was especially enriched in LGG^47^. Ro-4987655 is a novel mitogen-activated protein kinase kinase (MEK) inhibitor and currently under clinical development for cancer treatment^48^. Rociletinib, an epidermal growth factor receptor (EGFR) tyrosine kinase inhibitor widely used for non-small cell lung cancer patients, was highly abundant in 7 cancers especially in LIHC^49^. These findings suggest that a wide range of small molecule drugs, regardless of their clinical application status, possess the potential for broader therapeutic effects and warrant further in-depth exploration.

## Discussion

Glycosylation is a pivotal post-translational modification of proteins intricately regulated by various glycosyltransferases and glycosidases. Aberrant glycosylation is commonly observed in tumor cells and may result from alter expression of glycosidase enzymes^9^. PRKCSH is the non-catalytic subunit of glucosidase II that removes glucose from newly synthesized glycoproteins, generating monoglucosylated core oligosaccharides essential for protein folding^50,51^. At present, we have performed a systematic analysis of PRKCSH across 33 cancers using multiple public databases.

Our study identified PRKCSH mRNA expression abnormalities in most tumors. High PRKCSH expression was associated with poor prognosis in bladder, kidney, brain, lung, and sarcoma patients. PRKCSH may function as an oncogene influencing tumor progression and prognosis.

In recent decades, it has become evident that the interaction between non-cancerous cells and cancer cells in the TME can promote tumor growth throughout all stages of cancer development, thereby influencing early detection, treatment response, and patient prognosis^52^. The primary components of nonmalignant cells in the TME are infiltrating stromal and immune cells, and their proportions vary among patients with different cancer types. Then we further investigated the correlations between PRKCSH expression and the infiltration levels of 22 immune-related cells and found significant correlations between PRKCSH and multiple infiltrating immune cell types. Macrophages originating in monocytes have two different activated states that play opposite roles in tumor immunity^53,54^. Existing evidence suggests that M1 macrophages take part in the anti-tumor response^55^ not only by releasing inflammatory and chemokines to promote the inflammatory response^56^, but also by upregulating genes engaged in antigen processing, presentation, and costimulatory molecules, thereby enhancing T-cell response^57^. It is also recognized that M2 macrophages can be recruited to the TME by tumor cells and activated to create tumor-associated macrophages (TAM) that can promote tumor progression by enhancing genetic instability, angiogenesis, fibrosis, immunosuppression, lymphocyte exclusion, invasion, and metastasis^58^. Our study found a negative correlation between PRKCSH expression and M1 macrophage infiltration in 6 cancer types, and a positive correlation with M2 macrophage infiltration in 12 types of tumors. Mast cells have the ability to provide anti-tumor immunity by recruiting immune effector cells and enhancing immune surveillance^59^. The negative connections were also found between the activated mast cells and PRKCSH in 9 tumors. T cells, as the key components of adaptive immunity, also contribute to anti-tumor immunity. Cytotoxic T lymphocytes (CTLs), which are differentiated from CD8+ T cells and activated by antigen-presenting cells when faced with tumor cells, can directly kill tumor cells through releasing granules containing perforin, granzyme, and/or granulysin^60,61^, whereas CD4+ helper T cells (Th) secrete inflammatory factors such as IL-2, IL-4, IL-6, IFN-γ, etc., which can activate multiple immune cells (including CTLs, macrophages, B lymphocytes, and natural killer cells) to exert indirect anti-tumor effects and achieve tumor killing^62,63^. Our findings suggested negative correlations between PRKCSH expression and infiltration of CD4+ and CD8+ T cells in a variety of tumors. Based on this observation, we hypothesize that PRKCSH may protect cancer cells from being recognized and killed through inhibiting the proliferation of T cells. Dendritic cells, as representative antigen-presenting cells, have a pivotal role in the initiation and maintenance of antitumor T cell function^5^, and we observed a negative association between PRKCSH and dendritic cells in many tumors. In addition, correlation analysis on PRKCSH expression and immune molecules including checkpoints, immunostimulatory, immunoinhibitory, chemokines, and chemokines receptors showed that PRKCSH was positively related with these molecules in most tumors except in ACC, HNSC, KICH, KIRC, KIRP, LGG, LIHC, and PCPG. Taken together, these results cast illumination on the critical function of PRKCSH in regulating tumor immunity by interacting with TME, though further proof of experiments is required.

Our study identified genetic and epigenetic changes in PRKCSH as important mechanisms in carcinogenesis, with hypomethylation of PRKCSH associated with poor prognosis in some cancer types.

Then, we used PPI Network and GSEA analysis to explore the underlying mechanism of PRKCSH in tumorigenesis and cancer development. According to the GSEA analysis, PRKCSH was implicated in diverse cancer-linked immune pathways, mainly including adaptive immune response, response to chemokine, cytokine and so on, which reinforced our belief in PRKCH's immune role in tumors.

We conducted single-cell function analysis using CancerSEA and found that PRKCSH was associated with various functional states of cancer cells. PRKCSH may influence drug sensitivity and thus has potential as a therapeutic target. We also predicted small molecule drugs targeting PRKCSH using the Cmap database, some of which have already been used in tumor treatment or prevention.

The current study is a comprehensive and systematic bioinformatic analysis based on data from multiple databases. Additional assessment of these findings via in vitro and in vivo experimentation remains needed.

## Conclusion

PRKCSH is upregulated in a variety of tumors and associated with cancer prognosis. Notably, PRKCSH displays a correlation with TMB and MSI in certain tumors, indicating its potential as a predictive biomarker for immunotherapy response. PRKCSH is also involved in tumor immunity and correlated with various immune cells and immune molecules. PRKCSH may play a cancer-promoting role through genetic alteration and DNA methylation. Overall, our findings indicate that PRKCSH may represent a promising candidate for future tumor immunotherapy efforts.

### Supplementary Information

Supplementary Figures.

## Data Availability

All data generated or analyzed during this study are included in this article.
