# Supplementary material for: PRKCSH serves as a potential immunological and prognostic biomarker in pan-cancer
Source: Sci Rep. 2024 Jan 20;14:1778. doi: 10.1038/s41598-024-52153-w (PMC10799934; doi:10.1038/s41598-024-52153-w)
Supplement: Supplementary file 1 — Supplementary Figures. [file 41598_2024_52153_MOESM1_ESM.pdf]

Supplementary Materials for

PRKCSH Serves as a Potential Immunological and Prognostic Biomarker in Pan-cancer

Qiankun Wang[1], Xiong Wang[1], Jiaoyuan Li[1], Tongxin Yin[1], Yi Wang[1] and Liming Cheng[1*]

[1]Department of Laboratory Medicine, Tongji Hospital, Tongji Medical College, Huazhong University of Science and Technology, Wuhan 430030, China

*** Correspondence:**

Liming Cheng

chengliming2015@163.com

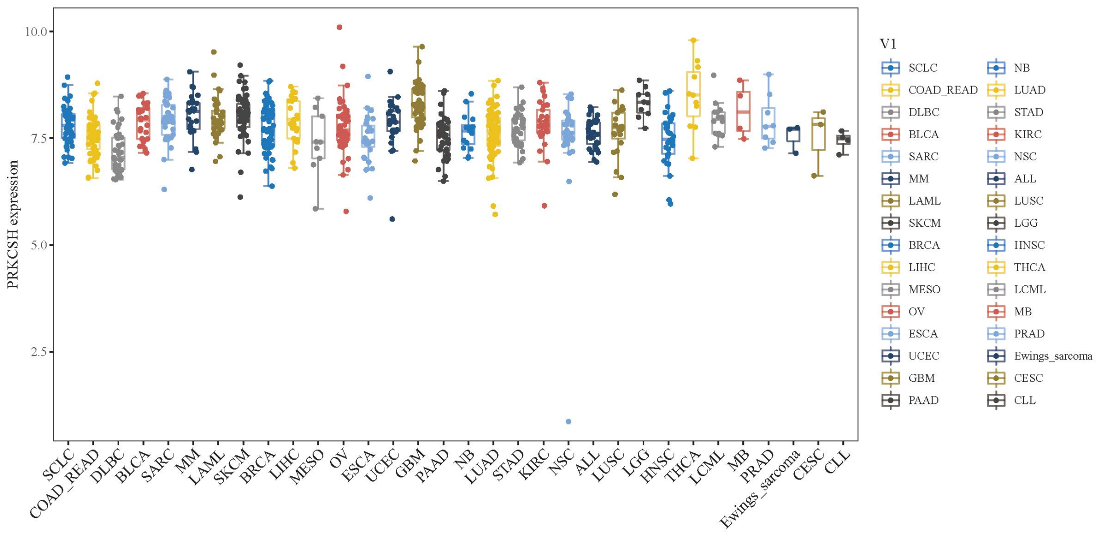

Figure S1 PRKCSH expression levels of 32 tumor cell lines in CCLE database.

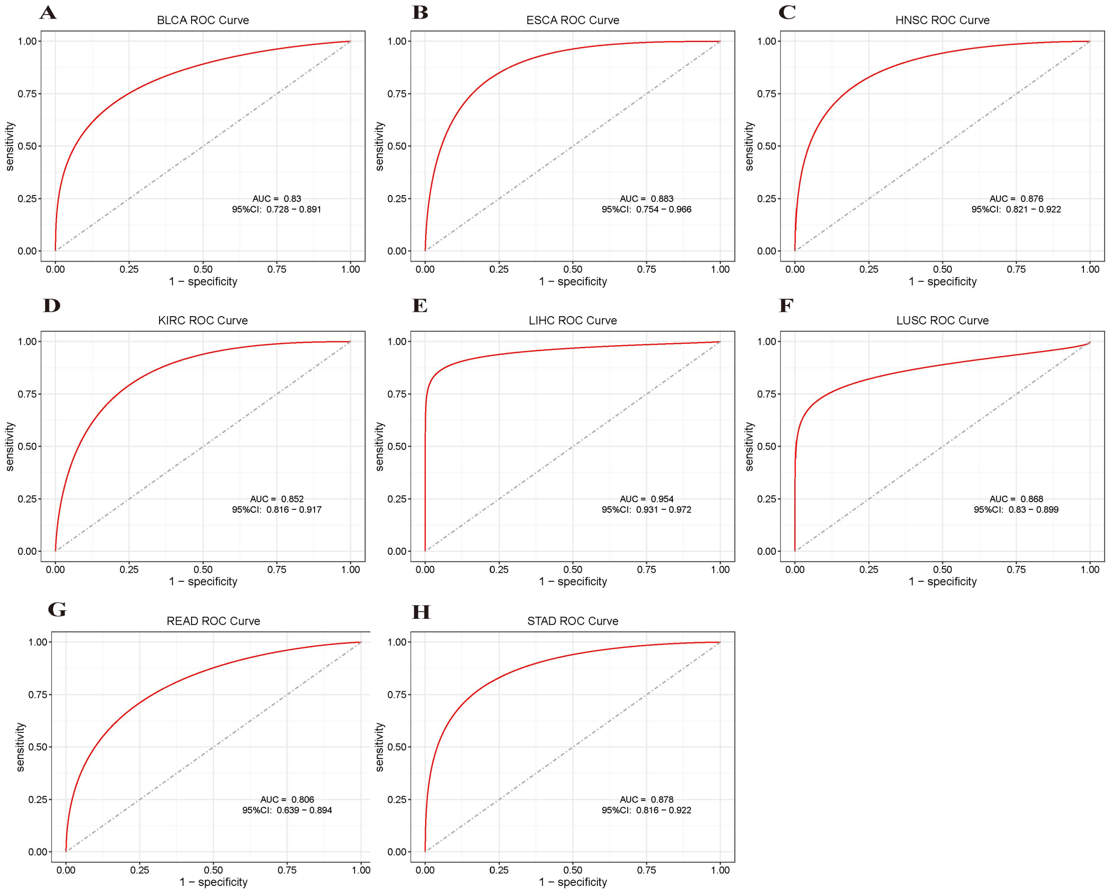

Figure S2 The diagnostic value of PRKCSH in pan-cancer. (A-H) The ROC curves of PRKCSH in BLCA, ESCA, HNSC, KIRC, LIHC, LUSC, READ, and STAD with AUC > 0.8.

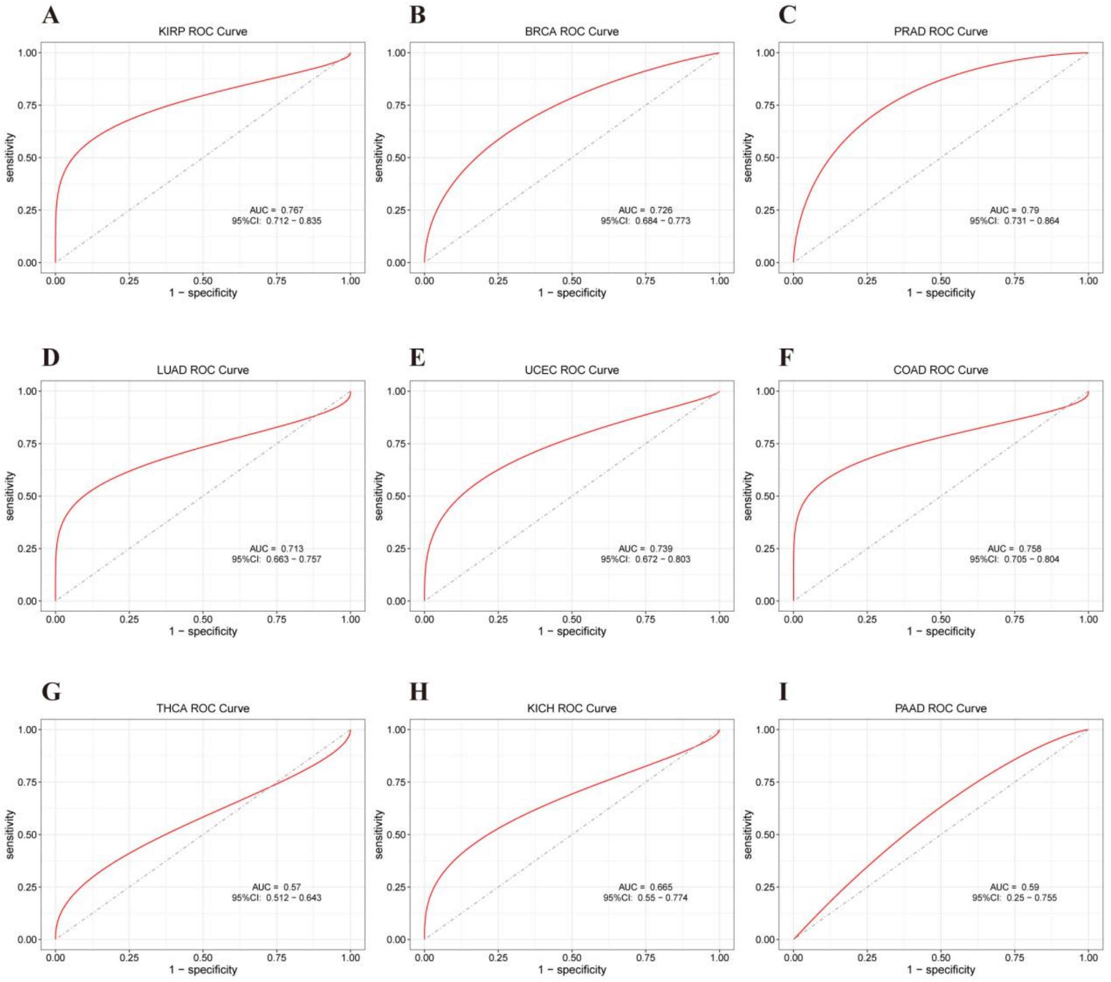

Figure S3 The diagnostic value of PRKCSH in pan-cancer. (A-I) The ROC curves of PRKCSH in KIRP, BRCA, PRAD, LUAD, UCEC, COAD, THCA, KICH, and PAAD with AUC < 0.8.

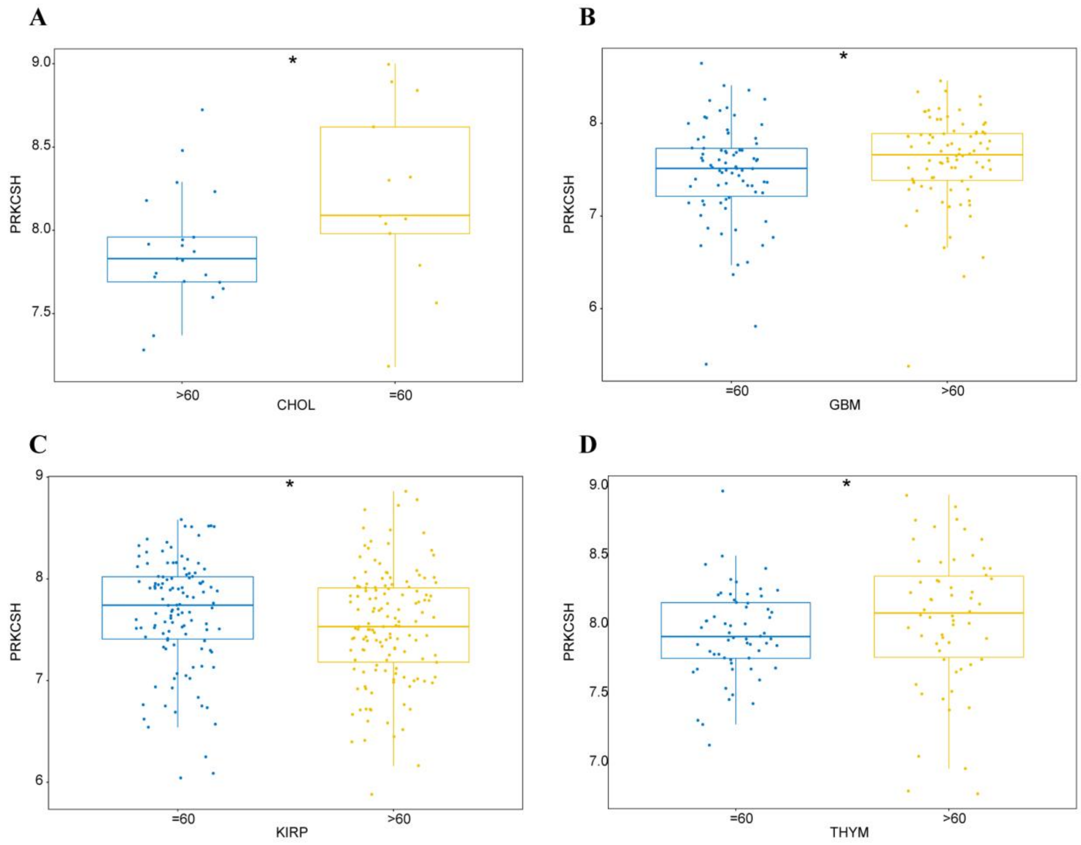

Figures S4 Correlation between PRKCSH expression and age in (A) CHOL, (B) GBM, (C) KIRP, and (D) THYM. *P < 0.05, **P < 0.01, and ***P < 0.001.

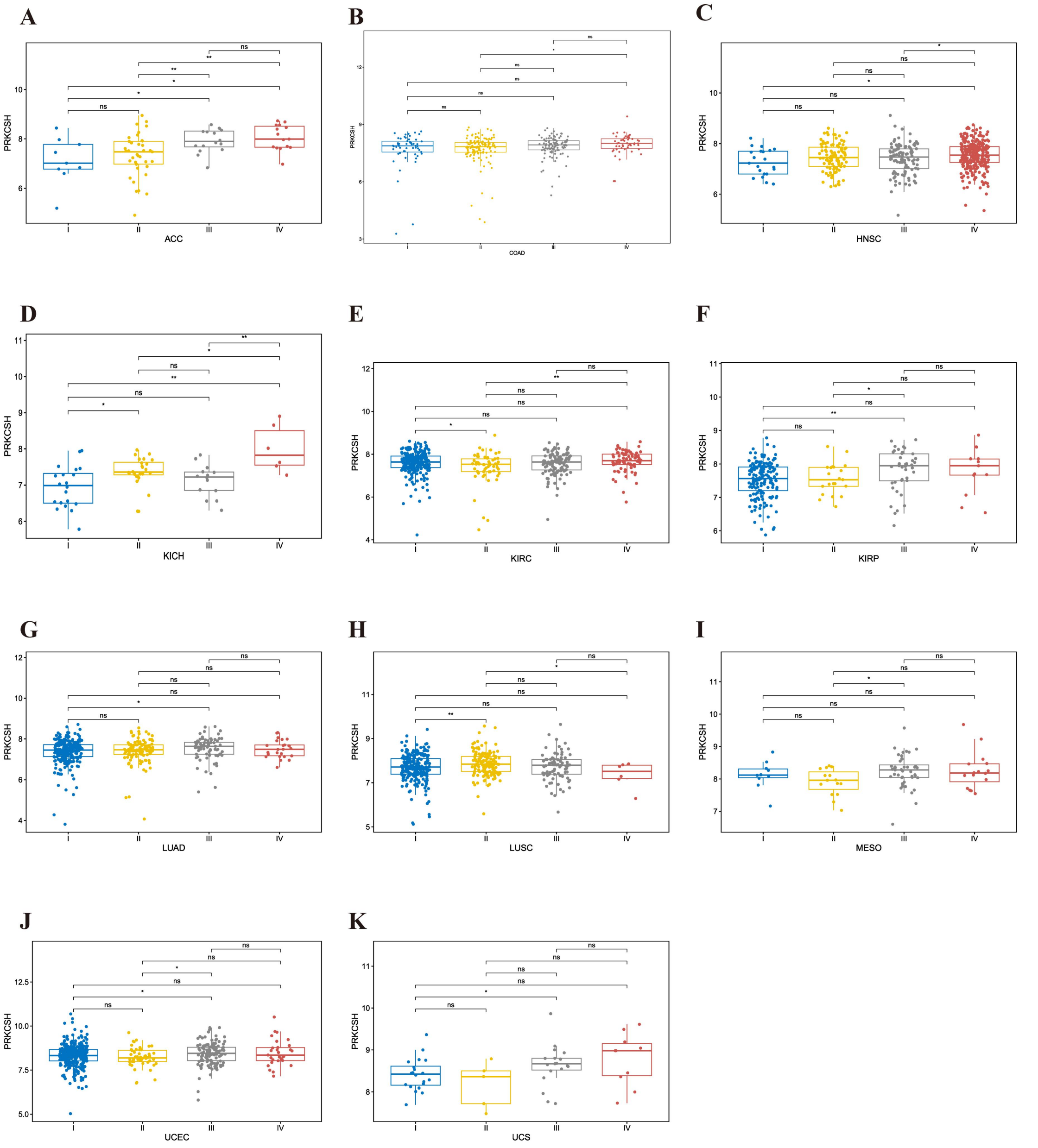

Figures S5 Correlation between PRKCSH expression and tumor stage in (A) ACC, (B) COAD, (C) HNSC, (D) KICH, (E) KIRC, (F) KIRP, (G) LUAD, (H) LUSC, (I) MESO, (J) UCEC, and (K) UCS. $^{*}P < 0.05$, $^{**}P < 0.01$, and $^{***}P < 0.001$.

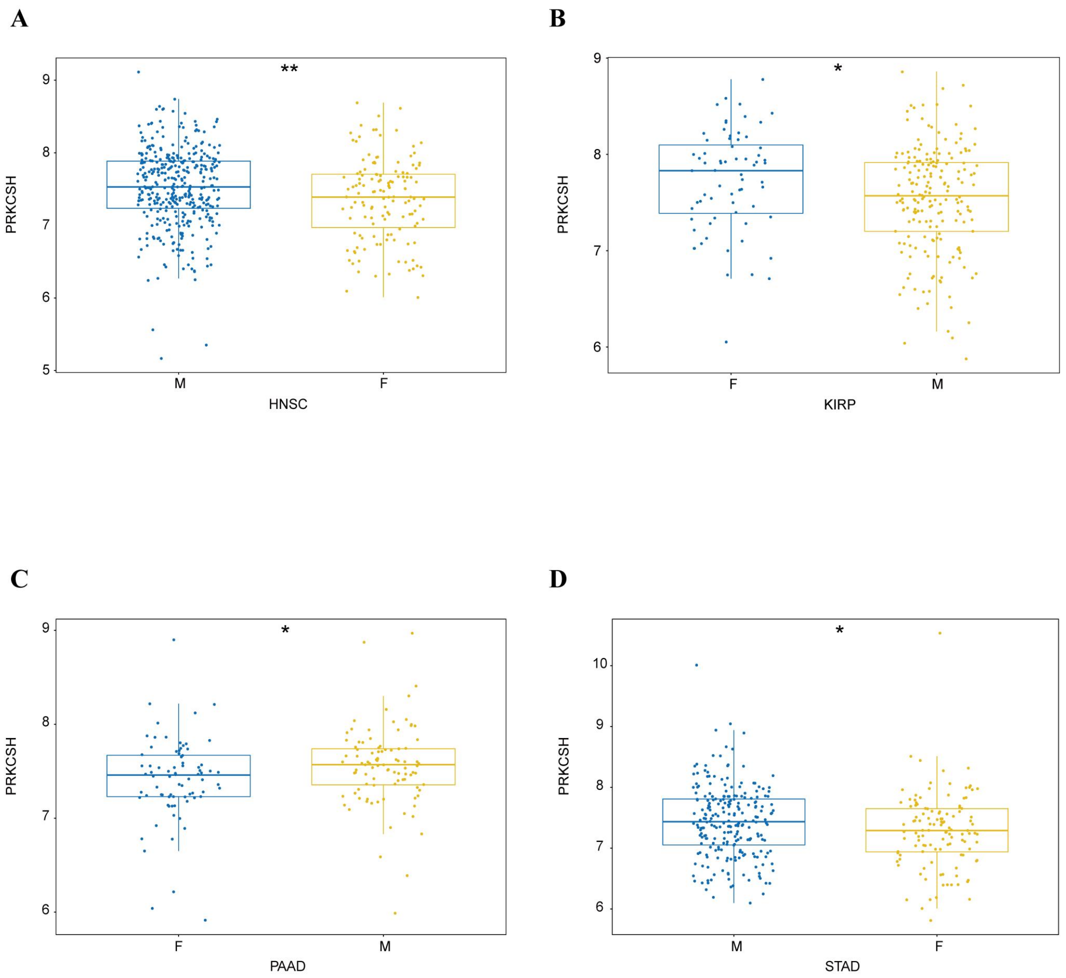

Figures S6 Correlation between PRKCSH expression and genders in (A) HNSC, (B) KIRP, (C) PAAD, and (D) STAD. *P < 0.05, **P < 0.01, and ***P < 0.001.

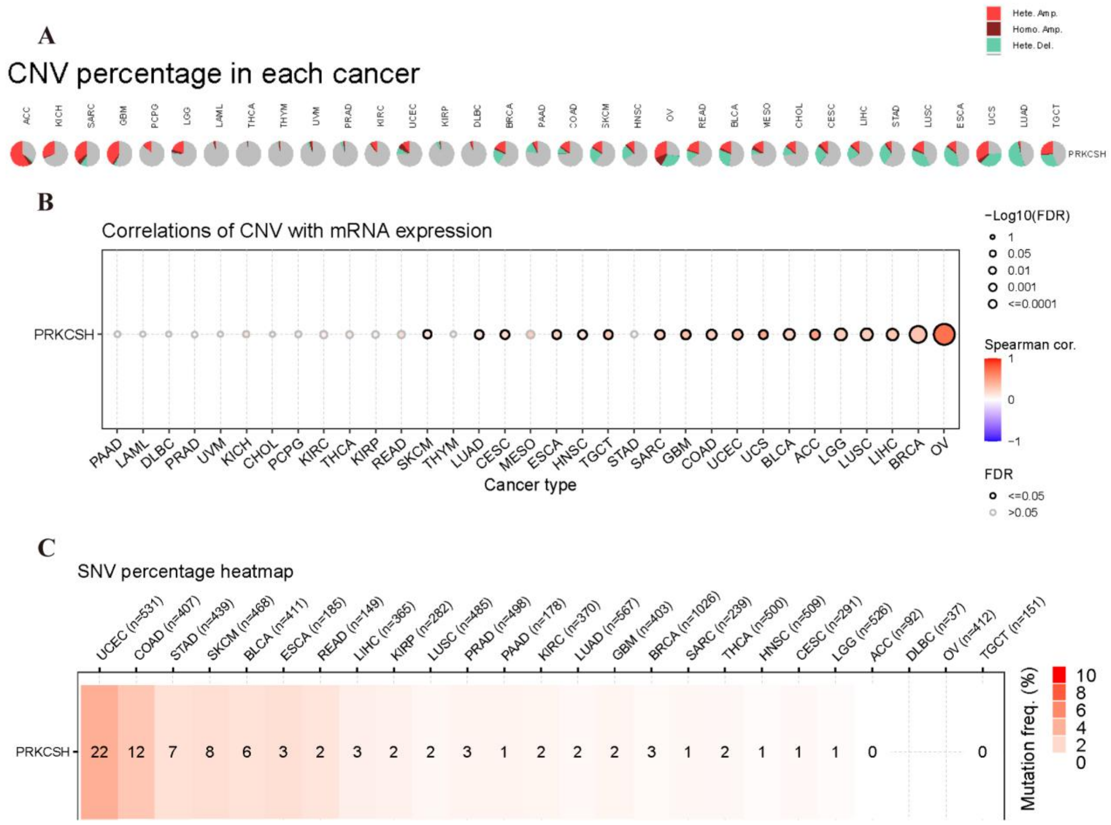

Figure S7 CNV and SNV analysis of PRKCSH by GSCA. (A) The CNV percentage of PRKCSH in each cancer. (B) The correlations between CNV and the expression of PRKCSH mRNA. (C) SNV percentage heatmap of PRKCSH in each cancer.

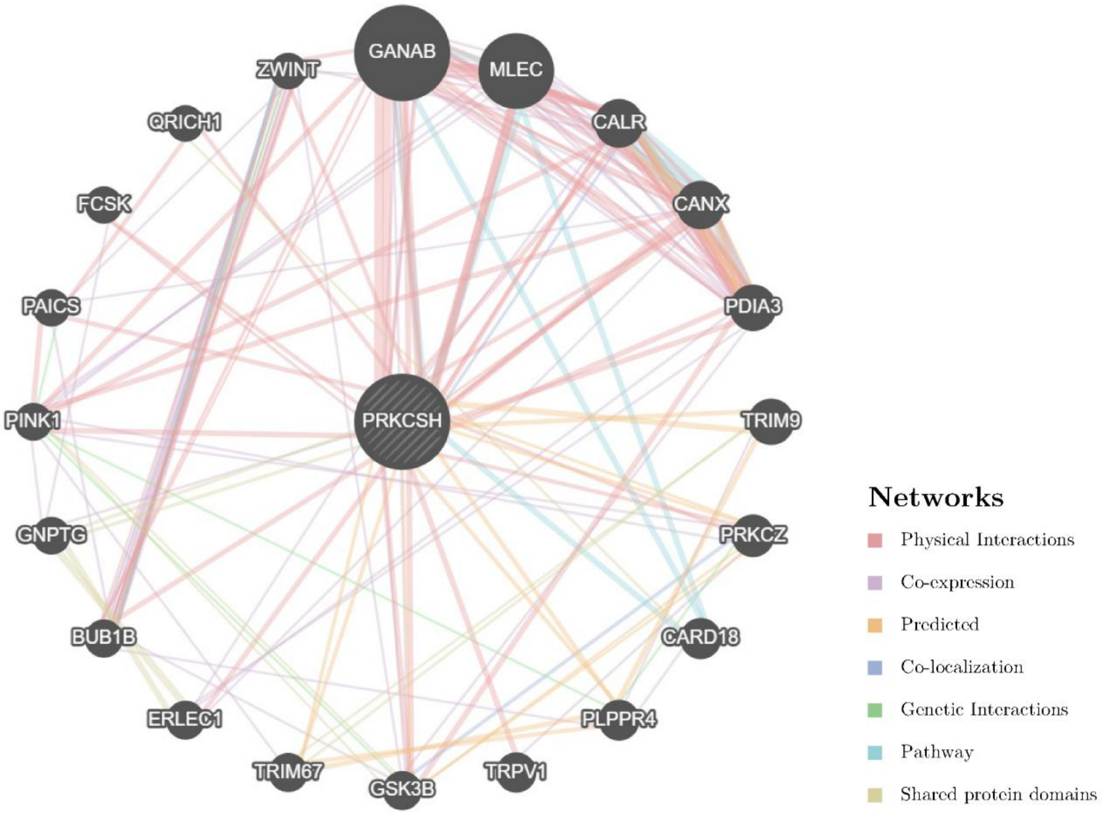

Figure S8 PPI network of PRKCSH constructed with GeneMANIA.

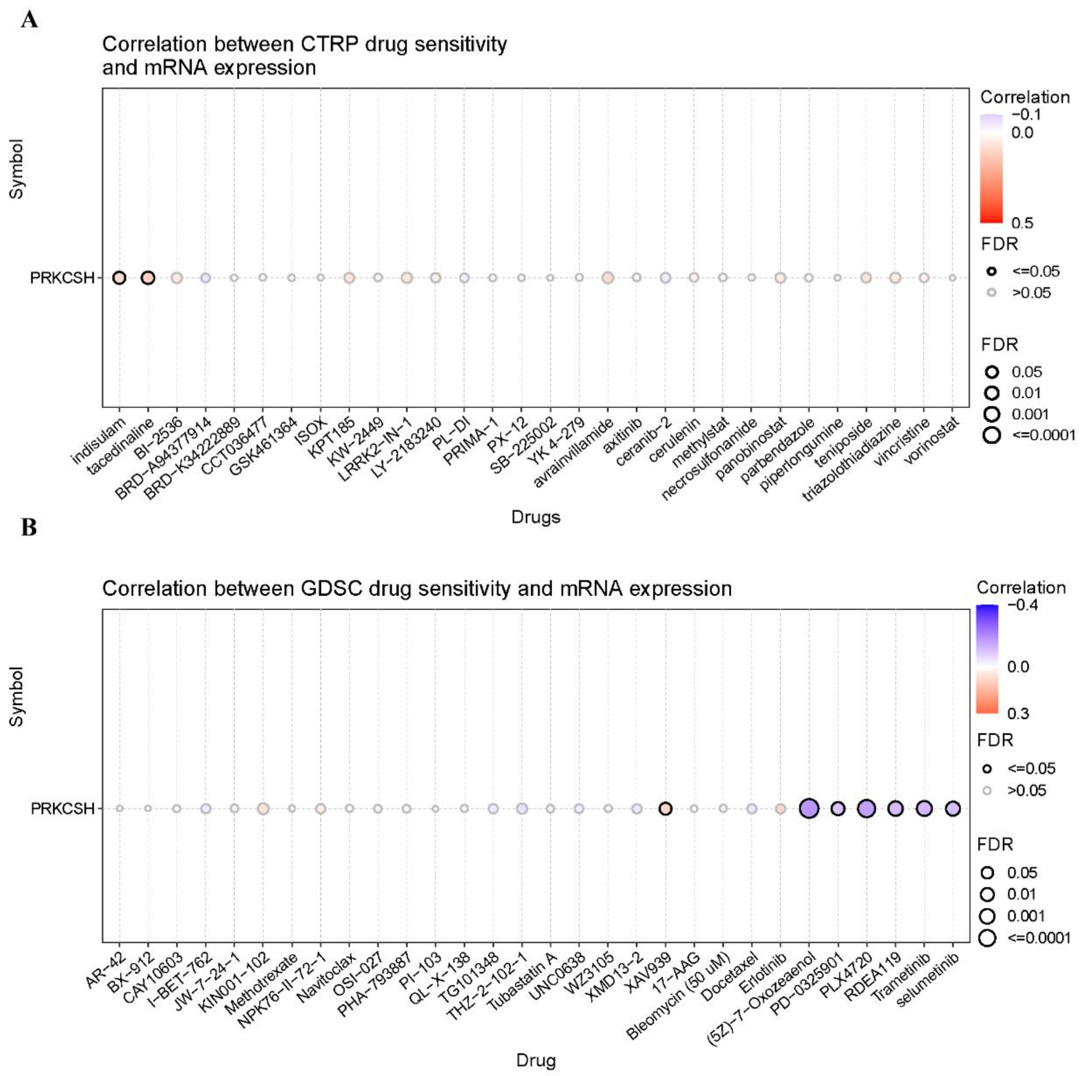

Figure S9 The analysis of drug sensitivity. PRKCSH correlates with drug sensitivity in (A) CTRP and (B) GDSC.
